# Supplementary material for: Main-chain engineering of polymer photocatalysts with hydrophilic non-conjugated segments for visible-light-driven hydrogen evolution
Source: Nat Commun. 2022 Sep 17;13:5460. doi: 10.1038/s41467-022-33211-1 (PMC9482619; doi:10.1038/s41467-022-33211-1)
Supplement: Supplementary file 1 — Supplementary Information [file 41467_2022_33211_MOESM1_ESM.pdf]

Supplementary Information

Main-chain engineering of polymer photocatalysts with hydrophilic non-conjugated segments for visible-light-driven hydrogen evolution

Chih-Li Chang[1,2], Wei-Cheng Lin[1], Li-Yu Ting[1], Chin-Hsuan Shih[3], Shih-Yuan Chen[4], Tse-Fu Huang[1], Hiroyuki Tateno[4], Jayachandran Jayakumar[1], Wen-Yang Jao[1], Chen-Wei Tai[1], Che-Yi Chu[5], Chin-Wen Chen[6], Chi-Hua Yu[3], Yu-Jung Lu[2], Chi-Chang Hu[1], Ahmed M. Elewa[1], Takehisa Mochizuki[4], and Ho-Hsiu Chou[1]*

[1]Department of Chemical Engineering, National Tsing Hua University, Hsinchu 300044, Taiwan

[2]Research Center for Applied Sciences, Academia Sinica, Taipei 115024, Taiwan

[3]Department of Engineering Science, National Cheng Kung University, Tainan 701401, Taiwan

[4]Energy Catalyst Technology Group, Energy Process Research Institute, National Institute of Advanced Industrial Science and Technology, Ibaraki 305-8559, Japan

[5]Department of Chemical Engineering, National Chung Hsing University, Taichung 402202, Taiwan

[6]Department of Molecular Science and Engineering, National Taipei University of Technology, Taipei 106344, Taiwan

*Corresponding author. E-mail: hhchou@mx.nthu.edu.tw

General methods: All reagents were obtained from commercial suppliers and were used without further purification. First, 2,7-Bis(4,4,5,5-tetramethyl-1,3,2-dioxaborolan-2-yl)-9,9-di-n-octylfluorene (F-B) and 3,7-dibromo-5-phenylbenzo[*b*]phosphindole-5-oxide (BPO-Br) were synthesized following procedures reported in our previous papers[1]. All reactions were performed under a nitrogen atmosphere with standard Schlenk techniques. ^{1}H, ^{13}C and ^{31}P NMR spectra were recorded in solution at 500 MHz, using a Bruker Advance 500 MHz NMR spectrometer. Mass spectra were obtained using a JEOL JMS-700 HRMS instrument. Fourier-transform infrared (FT-IR) spectra were recorded using a Thermo Scientific iS50 FT-IR infrared spectrometer. The molecular weights of the polymers were collected using a Hitachi gel permeation chromatograph and RI detector with polystyrene standards; the mobile phase was tetrahydrofuran (flow rate: 1 mL min^{-1}) at 40 °C. Thermogravimetric analysis of the polymers was performed under nitrogen using a TA Q600 instrument over the temperature range of 50–800 °C (heating rate: 10 °C min^{-1}). Powder X-ray diffraction (PXRD) measurements were performed using a Bruker Instruments D2-Phaser equipped with Cu Kα radiation. The HOMO energy levels were measured using a photoelectron spectrometer (model AC-II). UV–vis absorption spectra of the polymers were recorded on a Hitachi U-3300 spectrophotometer. Photoluminescence spectra of the polymers were recorded using a Hitachi F-7000 spectrophotometer. The optical bandgap (E_g) was obtained from Tauc plots ($(\alpha h\nu)^2$ versus $(h\nu)$) of the UV-Vis spectra and by extrapolation of the linear part of the curve to the energy axis based on the relationship $\alpha h\nu = A(h\nu - E_g)^\gamma$, where α is the absorption coefficient, A is an energy-independent constant, E_g is the optical band gap, h is Planck's constant, ν is the velocity, and γ is a constant representing the type of electronic transition. The LUMO energy levels were calculated by subtracting the E_g from the HOMO energy levels. Time-resolved transient PL decay spectra of the polymer photocatalysts were recorded on a spectrometer (FLS980, Edinburgh Instruments) with a gated photomultiplier tube. Charge generation was identified with an electron paramagnetic resonance spectrometer (EPR, Bruker E-580, X-band frequency) equipped with a Bruker ELEXSYS super-high-sensitivity cavity. A Hg lamp (400 W) with a major output at a wavelength of 365 nm was used to excite the photocatalysts at a fixed distance from the sample cavity. The measurements were set at a centre field of 3500 G and sweep width of 100 G. The microwave frequency was 9.49365 GHz and the power was 15.0 mW. Cyclic voltammetry (CV), electrochemical

impedance spectroscopy (EIS), and transient photocurrent measurements were performed using a Zahner Zennium E workstation equipped with a three-electrode cell. The hydrodynamic diameter of the polymer was measured using a Zetasizer Nano ZS90 (Malvern Instruments Nordic AB). SEM images were obtained using a Hitachi SU8010 scanning electron microscope operated at an acceleration voltage of 10 kV and used to observe the sample morphology. DB-FIB images were obtained using a FEI Helios Nanolab 600i System operated at an acceleration voltage of 2 kV and used to observe the thickness of polymer films. Water contact angle measurements were performed using a First Ten Angstroms FTA1000B surface contact angle analyser at 25 °C. Water for the H_2 evolution experiments was purified using an ELGA LabWater system. The apparent quantum yields (AQYs) for the photocatalytic H_2 evolution experiments were determined using the KIT-XENON-ADJ350W light source with a band-pass filter at centre wavelengths of 420, 460, 500, 550, and 600 nm for P-10H. Then, the AQY was calculated using following equation:

$$\boldsymbol{AQY}(\%) = \frac{\mathbf{2} \times \boldsymbol{Number\ of\ evolved\ H_2\ molecules}}{\boldsymbol{Number\ of\ incident\ photons}} \times \mathbf{100}\%$$

$$\frac{\mathbf{2} \times \boldsymbol{C} \times \boldsymbol{N_A}}{\boldsymbol{S} \times \boldsymbol{P} \times \boldsymbol{t} \times \frac{\boldsymbol{\lambda}}{(\boldsymbol{h} \times \boldsymbol{c})}} \times \mathbf{100}\%$$

where C is the amount of H_2 evolution per hour (mol), N_A is Avogadro constant (6.022×10^{23}/mol), S is the irradiation area (cm^2), P is the incident monochromatic light intensity (W/cm^2), t is the irradiation time (s), λ is the monochromatic light wavelength (m), h is Planck's constant (6.626×10^{-34} J·s), and c is the velocity of light in vacuum (3×10^8 m/s).

Synthesis procedures of co-monomers:

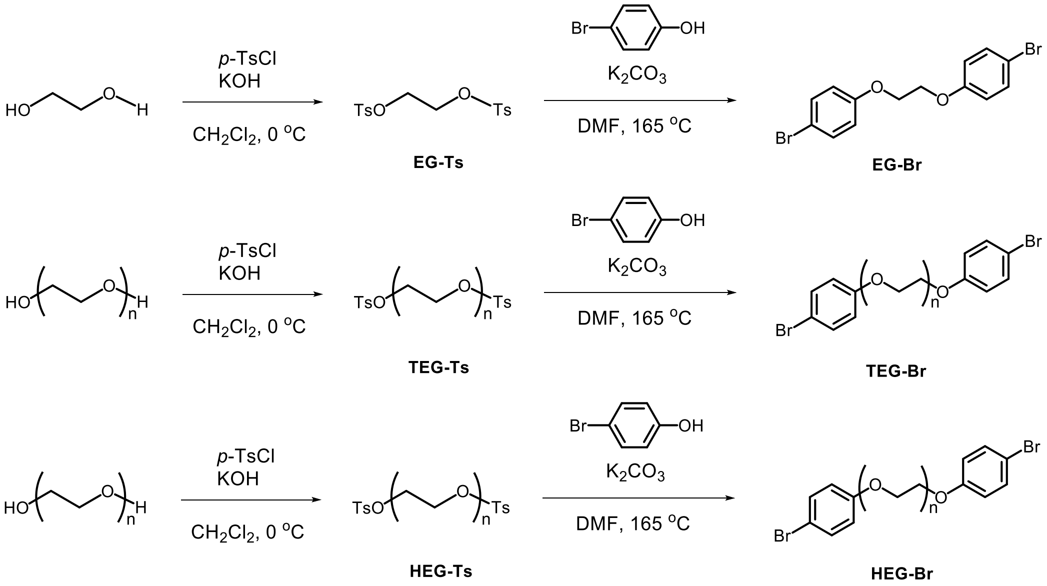

Synthesis of 1,2-bis(4-bromophenoxy)ethane (EG-Br):

A solution of ethylene glycol (1.115 mL, 20 mmol), 4-toluenesulfonyl chloride (8.389 g, 44 mmol), and potassium hydroxide (8.978 g, 160 mmol) in DCM (20 mL) was stirred at 0 °C for 3 h. The mixture was poured into water, extracted with DCM, and the organic layer was dried over anhydrous $MgSO_4$. The solvent was removed under reduced pressure and the product was obtained as a white powder (**EG-Ts**, Yield: 92.7%). To a two-neck reaction bottle (100 mL) containing **EG-Ts** (3.704 g, 10 mmol), 4-bromophenol (3.806 g, 22 mmol), and K_2CO_3 (6.081 g, 44 mmol) in DMF (40 mL) were added, and then the bottle was evacuated and degassed under nitrogen for 30 min at 25 °C. The reaction mixture was heated to 80 °C and stirred for 24 h. Then, the mixture was cooled to 25 °C, poured into H_2O, and extracted with EA. The combined organic layer was washed with brine, dried over anhydrous $MgSO_4$, and concentrated. The residue was purified by silica-gel column chromatography to extract the product **EG-Br** as a white solid (**EG-Br**, Yield: 74.2%). ^{1}H NMR (500 MHz, $CDCl_3$): δ 7.39 (d, J = 4.5 Hz, 4H), 6.83 (d, J = 4.5 Hz, 4H), 4.28 (s, 4H). ^{13}C NMR (500 MHz, $CDCl_3$): δ 157.64, 132.29, 116.46, 113.37, 66.65. HRMS (EI, m/z): [M^+] calcd for $C_{14}H_{12}Br_2O_2$: 369.9204; found, 369.9210.

Synthesis of 1,2-bis(2-(4-bromophenoxy)ethoxy)ethane (TEG-Br):

The procedure was same as that for EG-Br, except that ethylene glycol was replaced by triethylene glycol (2.672 mL, 20 mmol). The product **TEG-Br** was obtained as a white solid (Yield: 71.9%). ^{1}H NMR (500 MHz, $CDCl_3$): δ 7.35 (d, J = 4.5 Hz, 4 H), 6.78 (d, J = 4.5 Hz, 4 H), 4.08 (t, J = 4.5 Hz, 4 H), 3.85 (t, J = 4.5 Hz, 4 H), 3.73 (s, 4 H). ^{13}C NMR (500 MHz, $CDCl_3$): δ 157.82, 132.15, 116.38, 112.96, 70.80, 69.62, 67.60. HRMS (EI, m/z): [M^+] calcd for $C_{18}H_{20}Br_2O_4$: 457.9728; found, 457.9727.

Synthesis of 1,17-bis(4-bromophenoxy)-3,6,9,12,15-pentaoxaheptadecane (HEG-Br):

The procedure was same as that for EG-Br except that ethylene glycol was replaced by hexaethylene glycol (5.028 mL, 20 mmol). The product **HEG-Br** was obtained as a yellow-orange oil (Yield: 68.6%). ^{1}H NMR (500 MHz, $CDCl_3$): δ 7.35 (d, J = 4.5 Hz, 4 H), 6.78 (d, J = 4.5 Hz, 4 H), 4.08 (t, J = 4.5 Hz, 4 H), 3.83 (t, J = 4.5 Hz, 4 H), 3.71-3.65 (m, 4 H), 3.64 (s, 12 H). ^{13}C NMR (500 MHz, $CDCl_3$): δ 157.68, 131.96, 116.25, 112.72, 70.57, 70.35, 70.31, 69.36, 67.44. HRMS (EI, m/z): [M^+] calcd for $C_{24}H_{32}Br_2O_7$: 590.0515; found, 590.0517.

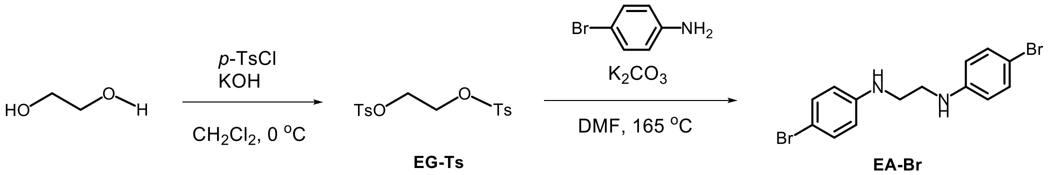

Synthesis of *N*^{1},*N*^{2}-bis(2-(4-bromophenyl)ethane-1,2,-diamine (EA-Br):

The procedure was same as that for EG-Br, except that ethylene glycol was replaced by ethylene diamine (4.94 mL, 20 mmol). The product **EA-Br** was obtained as a white solid (Yield: 60.9%). ^{1}H NMR (500 MHz, $CDCl_3$): δ 7.25 (d, J = 10 Hz, 4 H), 6.49 (d, J = 10 Hz, 4 H), 3.83 (s, 2 H), 3.33 (s 4 H). ^{13}C NMR (500 MHz, $CDCl_3$): δ 146.84, 132.06, 114.60, 109.60, 67.96. HRMS (EI, m/z): [M^+] calcd for $C_{18}H_{20}Br_2O_4$: 367.9524; found, 367.9529.

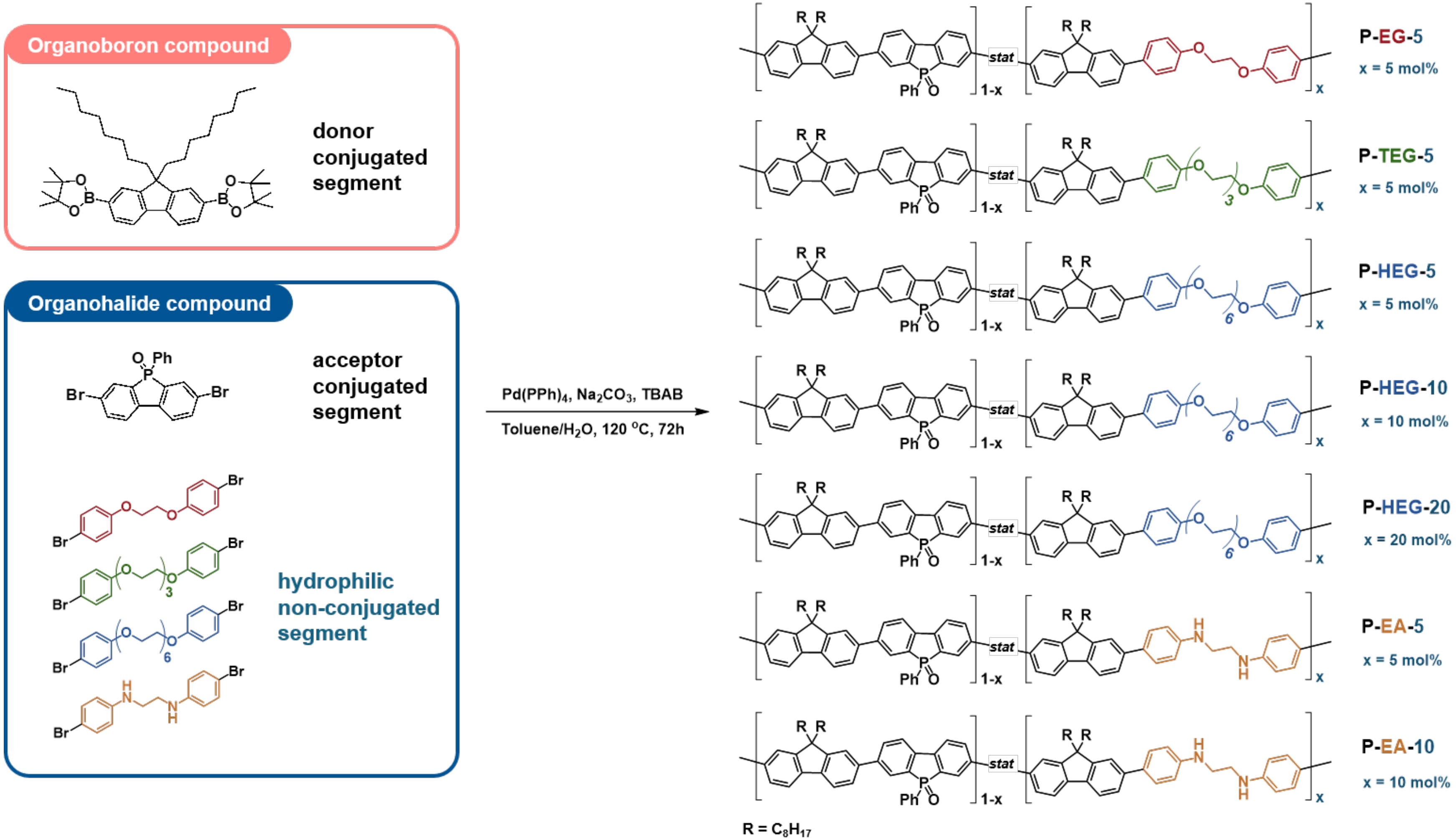

Supplementary Fig. 1 Synthesis procedure of polymer photocatalysts. Pd-catalyzed Suzuki−Miyaura coupling polymerization of fluorene-boronic ester and 3,7-dibromo-5-phenylbenzo[*b*]phosphindole-5-oxide with either EG-based (EG-Br, TEG-Br, and HEG-Br) or EA-based (EA-Br) segments is used to synthesize polymer photocatalysts.

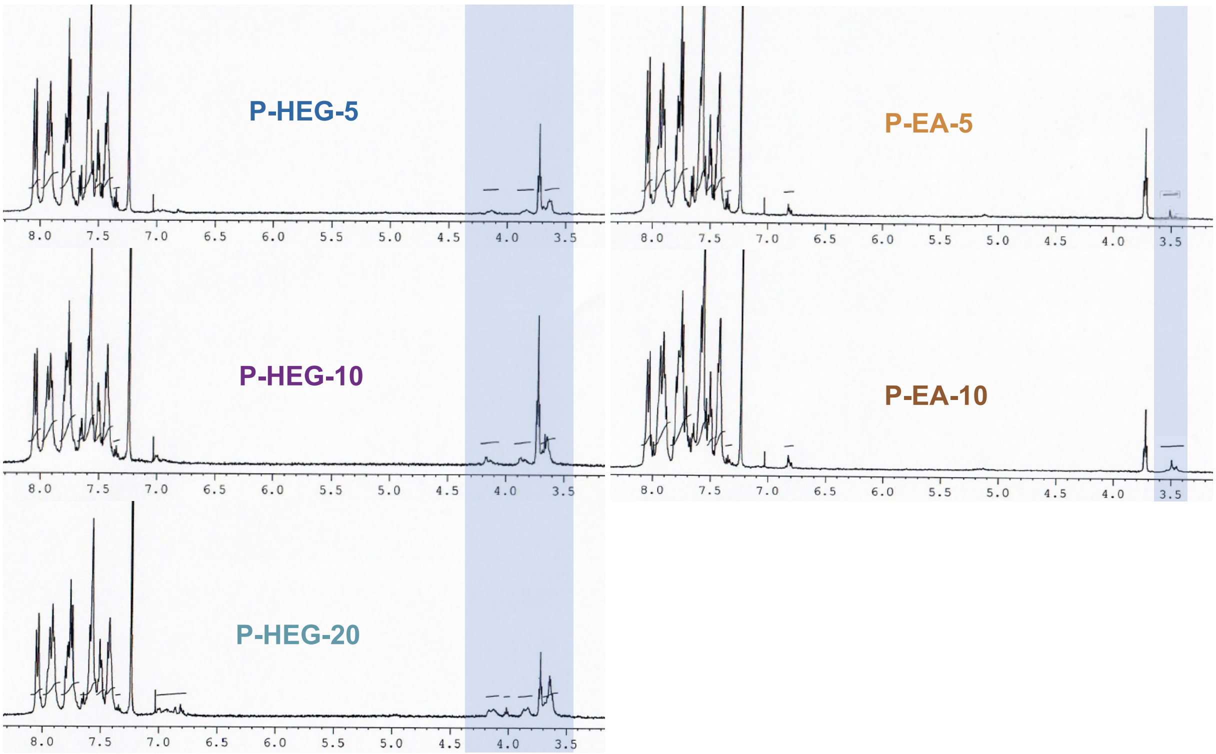

Supplementary Fig. 2 ^{1}H NMR characterization of polymers. The ratio of HEG and EA segments in the polymer backbone is determined from the characteristic signals of HEG and EA segments at 3.5–4.5 ppm in the ^{1}H NMR spectrum of the polymer.

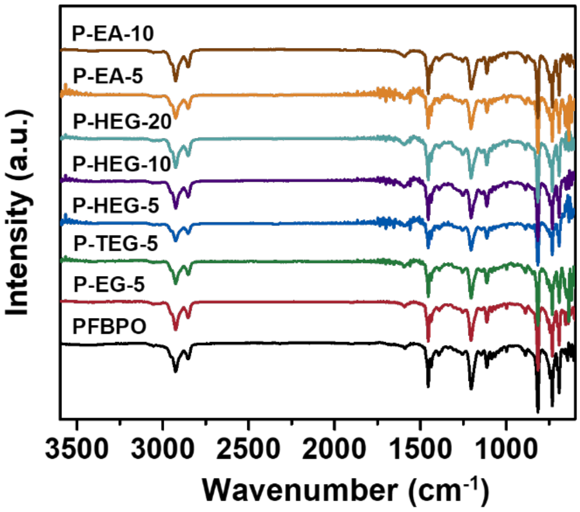

Supplementary Fig. 3 FT-IR characterization. FT-IR spectra of the polymers.

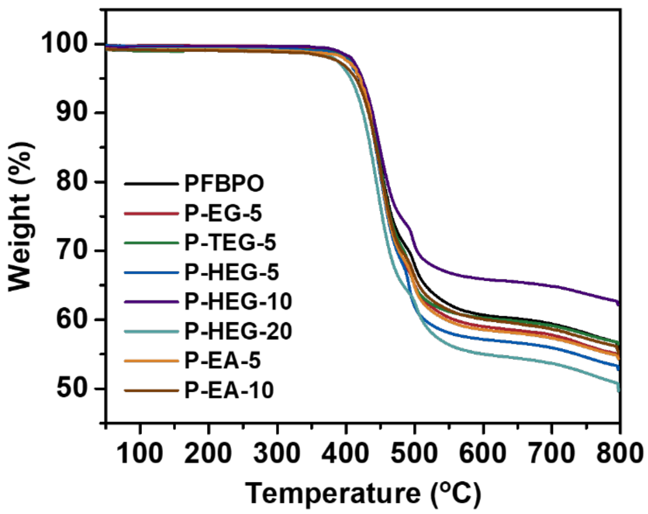

Supplementary Fig. 4 Thermogravimetry analysis. Thermogravimetry analysis of the polymers was performed under nitrogen over the temperature range of 50–800 °C (heating rate: 10 °C min^{-1}).

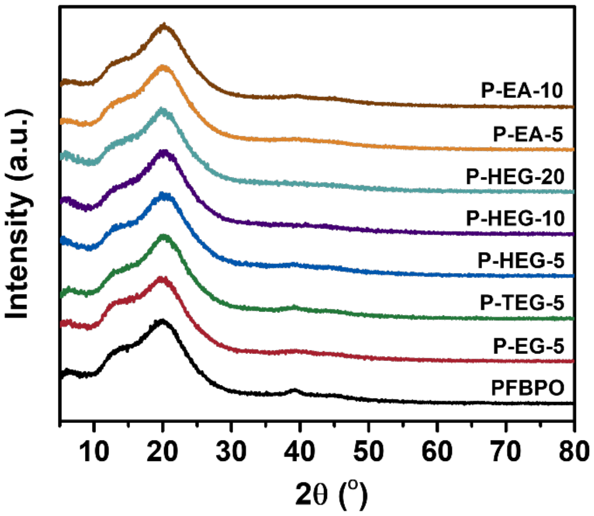

Supplementary Fig. 5 PXRD analysis. PXRD patterns of the polymers.

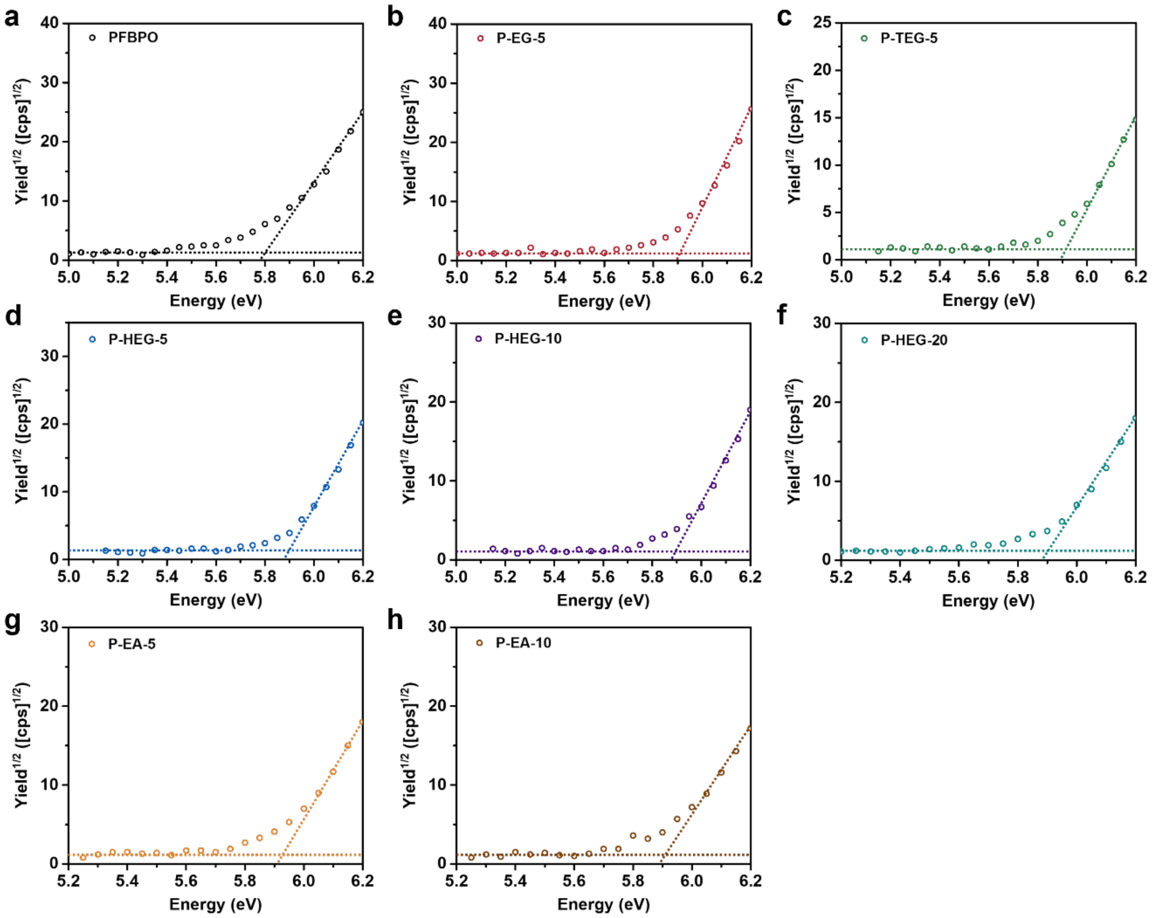

Supplementary Fig. 6 Photoelectronic analysis. Photoelectronic spectra of **a** PFBPO, **b** P-EG-5, **c** P-TEG-5, **d** P-HEG-5, **e** P-HEG-10, **f** P-HEG-20, **g** P-EA-5, and **h** P-EA-10 were performed to determine the HOMO levels.

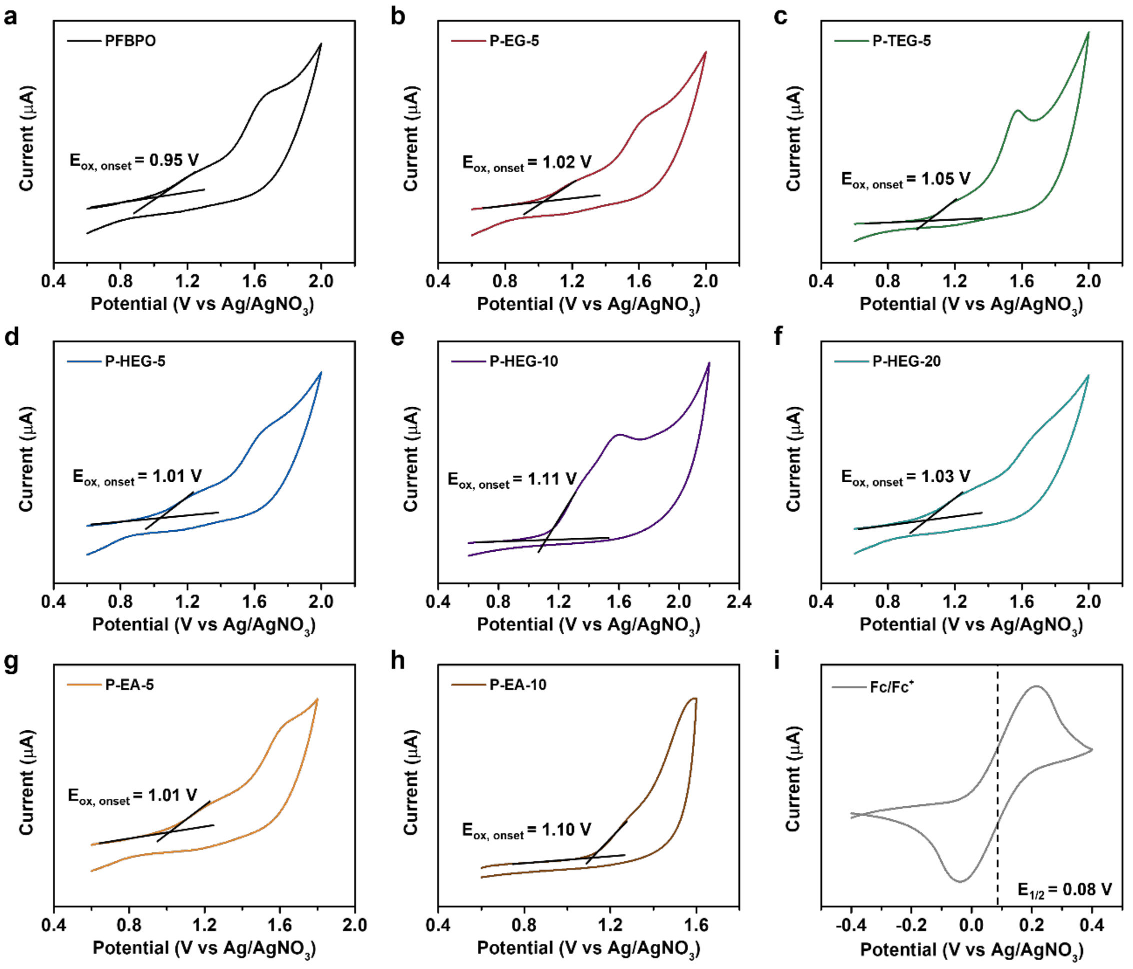

Polymer	Oxidation Potential (V vs NHE)	$E_{HOMO,CV}$ (eV)	$E_{HOMO,PESA}$ (eV)
**PFBPO**	1.27	−5.67	−5.82
**P-EG-5**	1.35	−5.74	−5.92
**P-TEG-5**	1.37	−5.77	−5.91
**P-HEG-5**	1.33	−5.73	−5.91
**P-HEG-10**	1.43	−5.83	−5.92
**P-HEG-20**	1.35	−5.75	−5.90
**P-EA-5**	1.34	−5.73	−5.93
**P-EA-10**	1.42	−5.82	−5.91

Supplementary Fig. 7 Cyclic voltammetry analysis. Cyclic voltammetry measurements of **a** PFBPO, **b** P-EG-5, **c** P-TEG-5, **d** P-HEG-5, **e** P-HEG-10, **f** P-HEG-20, **g** P-EA-5, **h** P-EA-10, and **i** ferrocene/ferrocenium couple. Ferrocene/ferrocenium (Fc/Fc^+) redox potential was measured under the same condition to calibrate the reference electrode. The HOMO levels are determined as follows: $E_{HOMO} = -(E_{ox,onset} - E\ (Fc/Fc^+) + 4.8)$ eV.

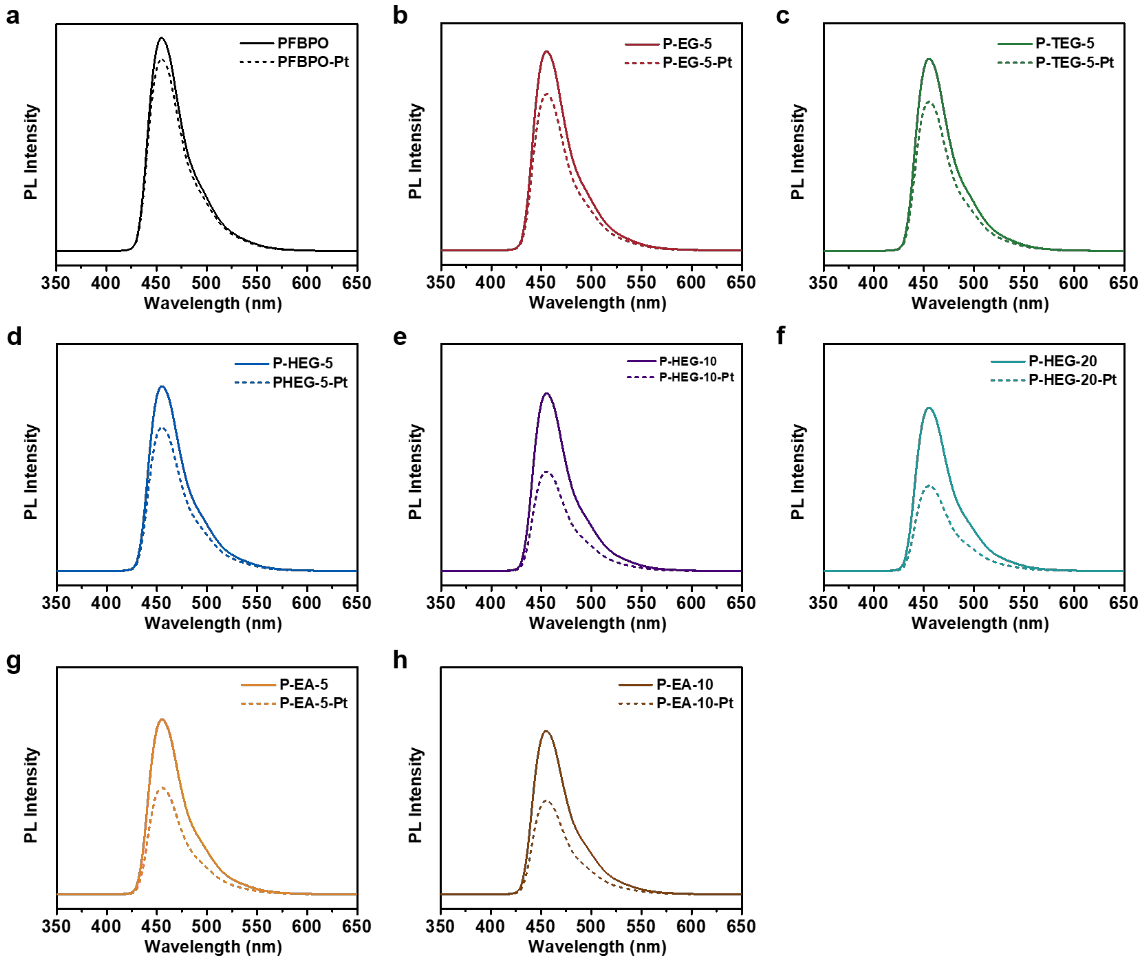

Supplementary Fig. 8 Photoluminescence characterization. Emission spectra of **a** PFBPO, **b** P-EG-5, **c** P-TEG-5, **d** P-HEG-5, **e** P-HEG-10, **f** P-HEG-20, **g** P-EA-5, and **h** P-EA-10 were obtained in suspensions (5 mg photocatalyst in 10 mL mixture consisting of equal volumes of H_2O, MeOH, and TEA) before (solid line) and after (dash line) adding 3 wt.% H_2PtCl_6 co-catalyst, respectively, in which the pH keeps consistent at 12.4.

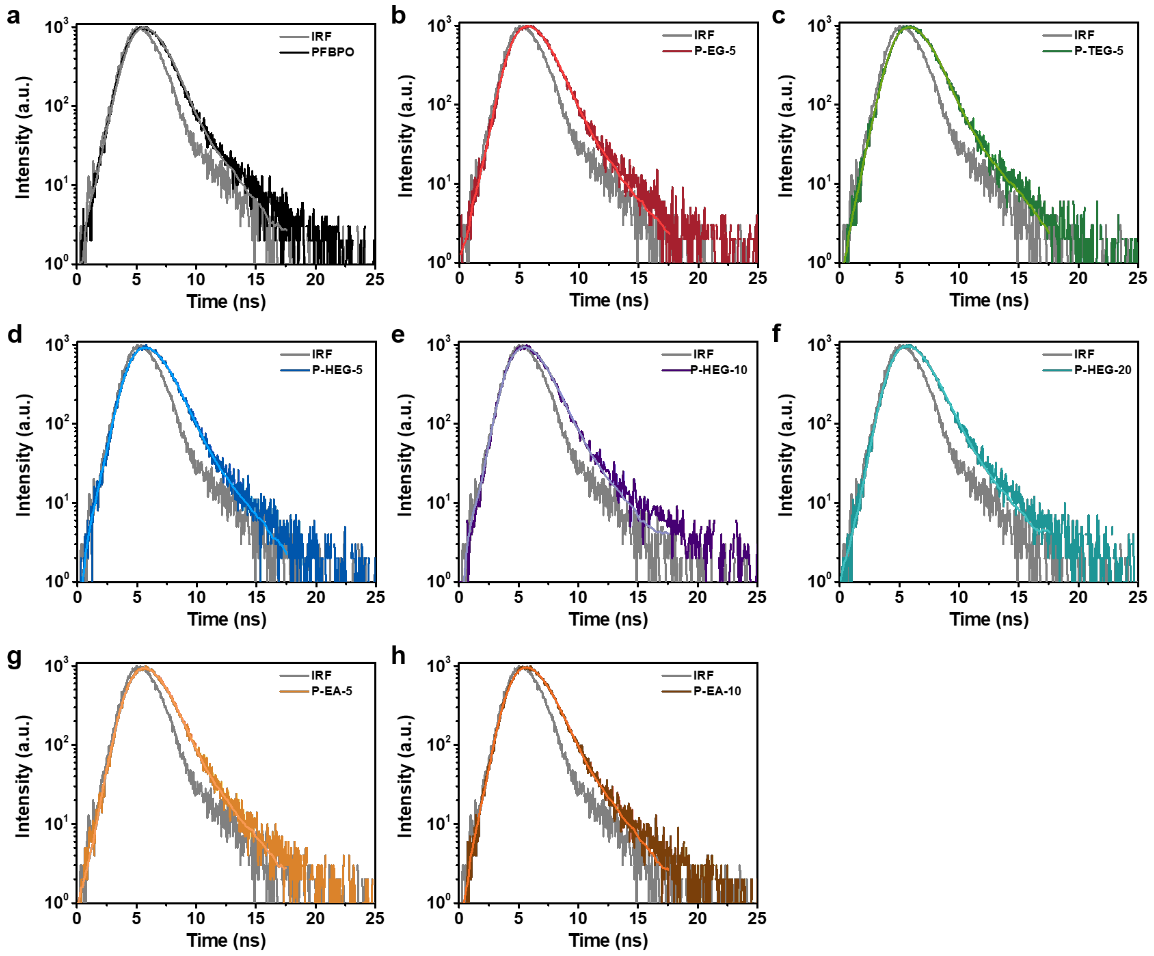

Supplementary Fig. 9 Time-resolved photoluminescence characterization. Time-resolved photoluminescence spectra of **a** PFBPO, **b** P-EG-5, **c** P-TEG-5, **d** P-HEG-5, **e** P-HEG-10, **f** P-HEG-20, **g** P-EA-5, and **h** P-EA-10 were obtained in suspensions (5 mg photocatalyst in 10 mL solution mixture consisting of equal volumes of H_2O, MeOH, and TEA). Samples were excited with a 405 nm laser and emission was measured at 455 nm.

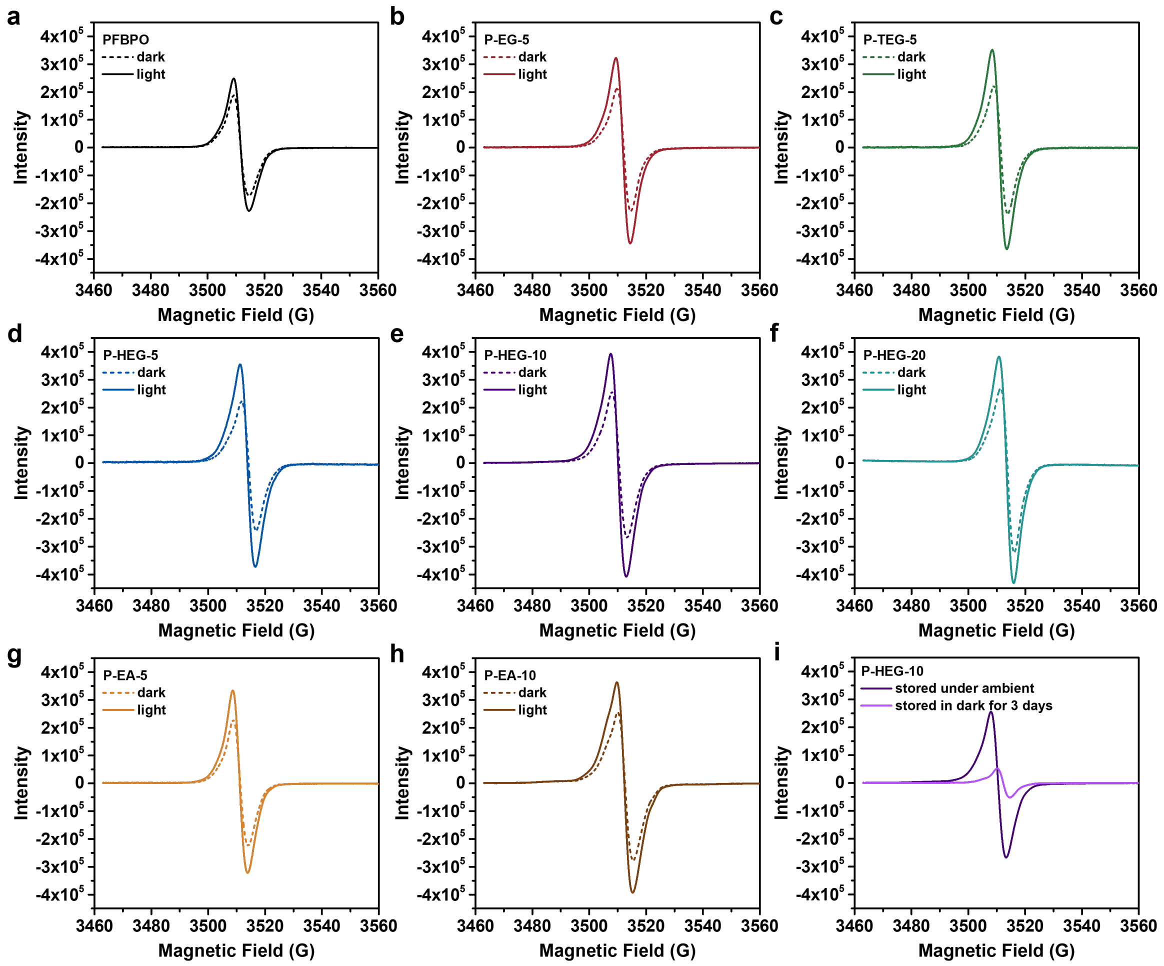

Supplementary Fig. 10 EPR characterization of polymers. EPR spectra of **a** PFBPO, **b** P-EG-5, **c** P-TEG-5, **d** P-HEG-5, **e** P-HEG-10, **f** P-HEG-20, **g** P-EA-5, and **h** P-EA-10 were measured without (dash line) and with (solid line) additional light irradiation and **i** EPR spectra of P-HEG-10 stored under ambient conditions and stored in the dark for 3 days.

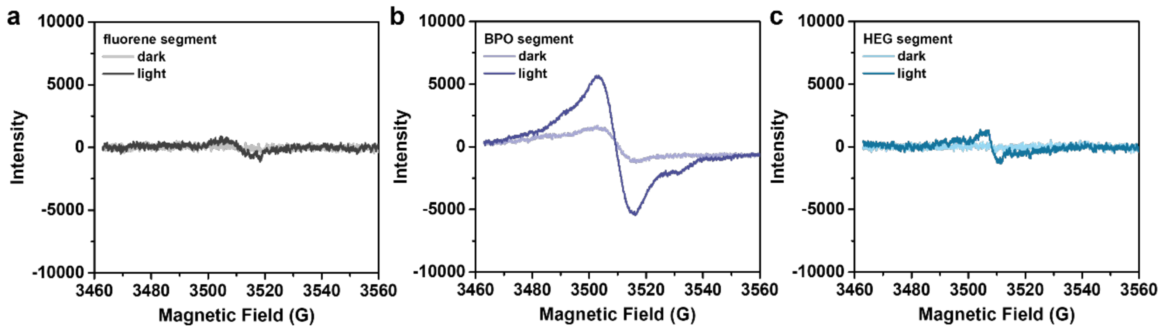

Supplementary Fig. 11 EPR characterization of co-monomers. EPR spectra of **a** fluorene, **b** BPO, **c** HEG segments were measured without and with additional light irradiation.

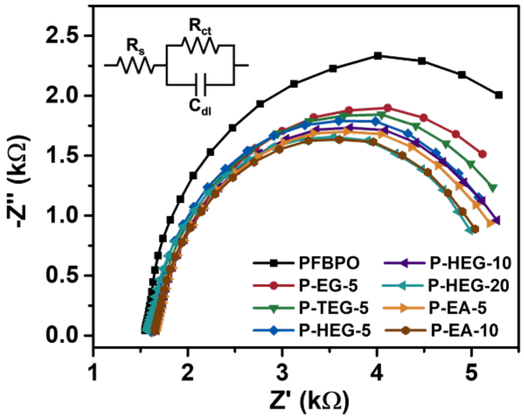

Polymer	R_s (Ω)	R_{ct} (Ω)
**PFBPO**	1552	5128
**P-EG-5**	1574	4668
**P-TEG-5**	1618	4436
**P-HEG-5**	1635	4252
**P-HEG-10**	1654	4104
**P-HEG-20**	1658	3942
**P-EA-5**	1668	4056
**P-EA-10**	1651	3984

Supplementary Fig. 12 Electrochemical impedance analysis. Electrochemical impedance spectra of polymers were carried out in dark with an AC potential frequency ranging from 0.1 Hz to 100 kHz. In the equivalent circuit (inset), R_s represents the circuit series-resistance, R_{ct} is the charge transfer resistance across the interface, and C_{dl} is the capacitance phase element of the semiconductor-electrolyte interface. Simulated R_s and R_{ct} values of polymers for electrochemical impedance test were list in table.

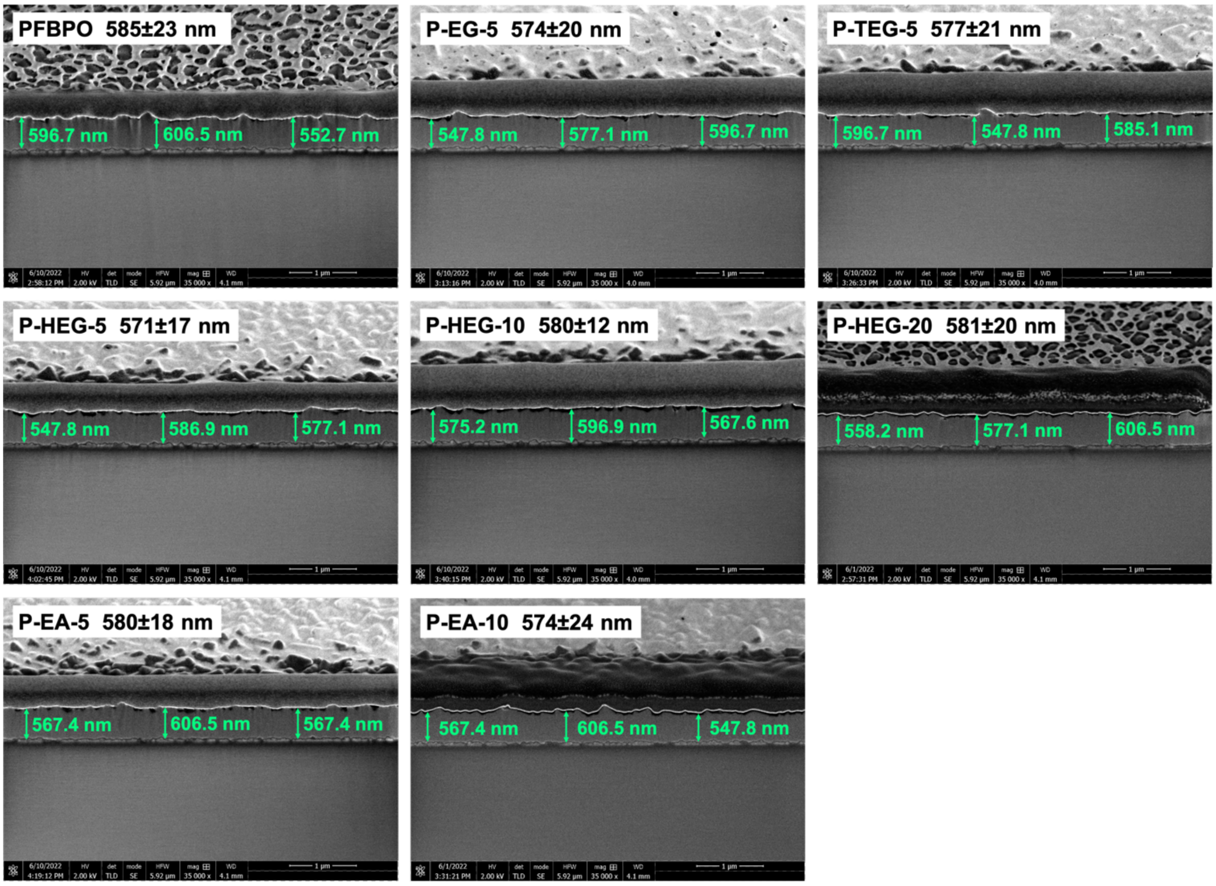

Supplementary Fig. 13 Thickness of polymer films. DB-FIB images of all polymers were used to measure polymer film thickness.

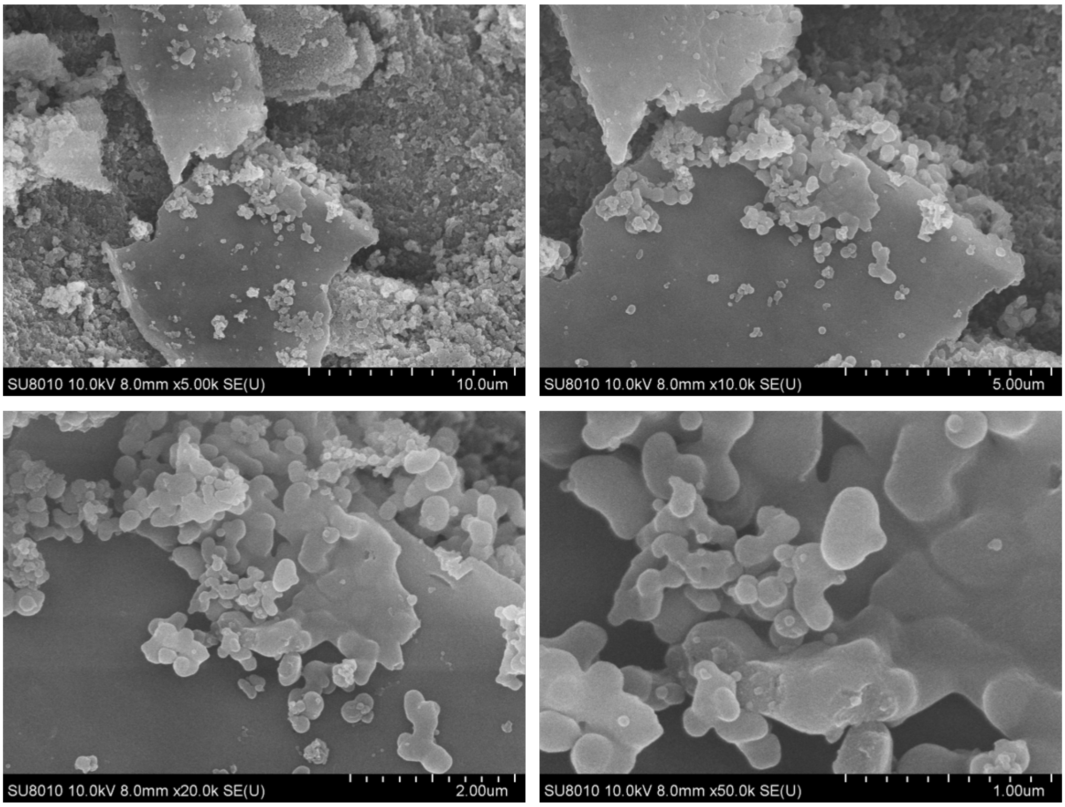

Supplementary Fig. 14 Morphologies of polymers. SEM images of PFBPO.

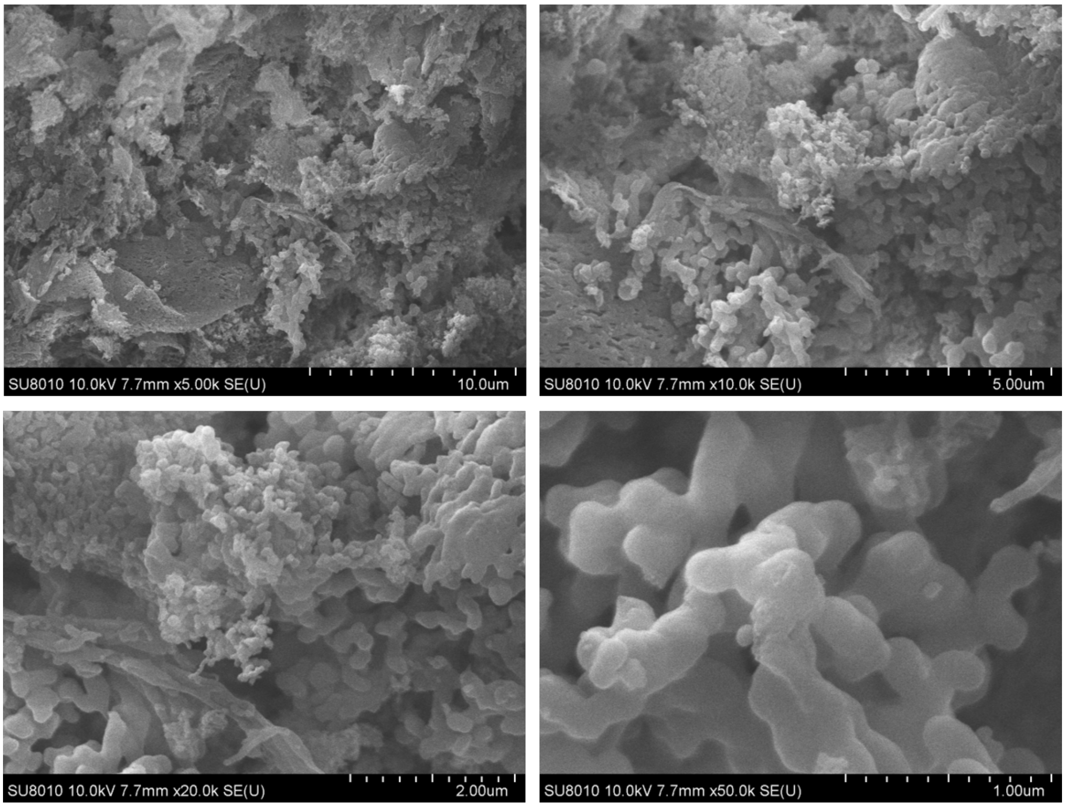

Supplementary Fig. 15 Morphologies of polymers. SEM images of P-EG-5.

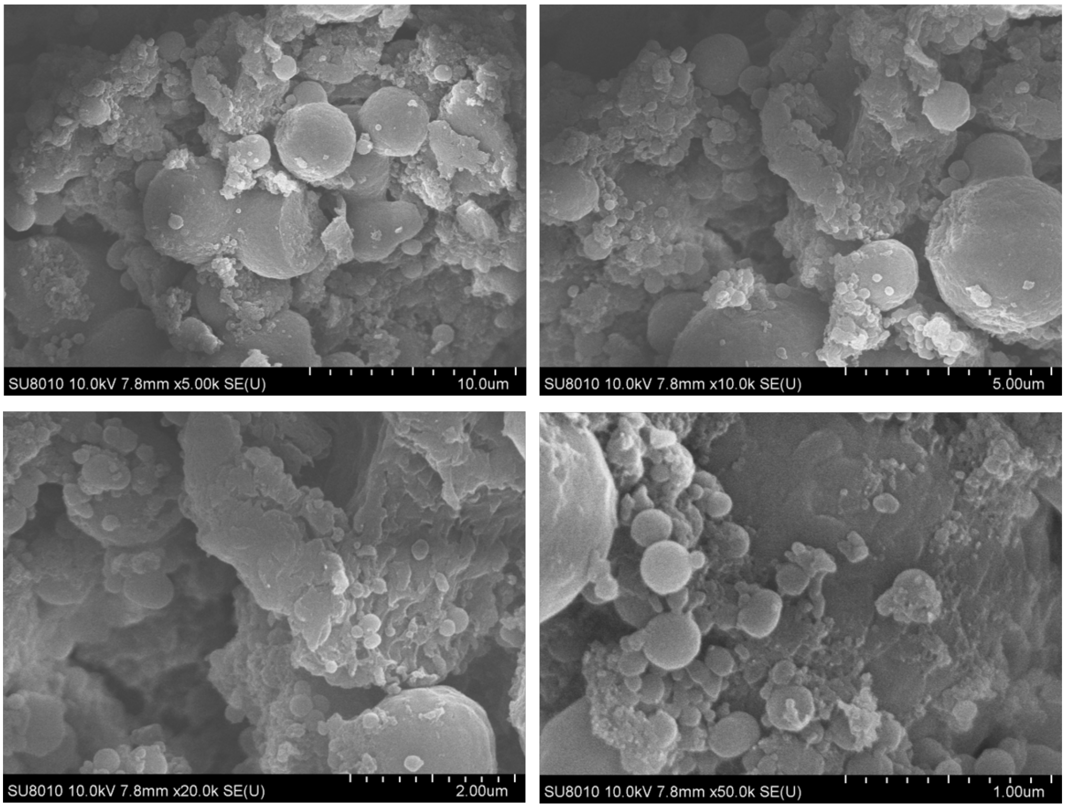

Supplementary Fig. 16 Morphologies of polymers. SEM images of P-TEG-5.

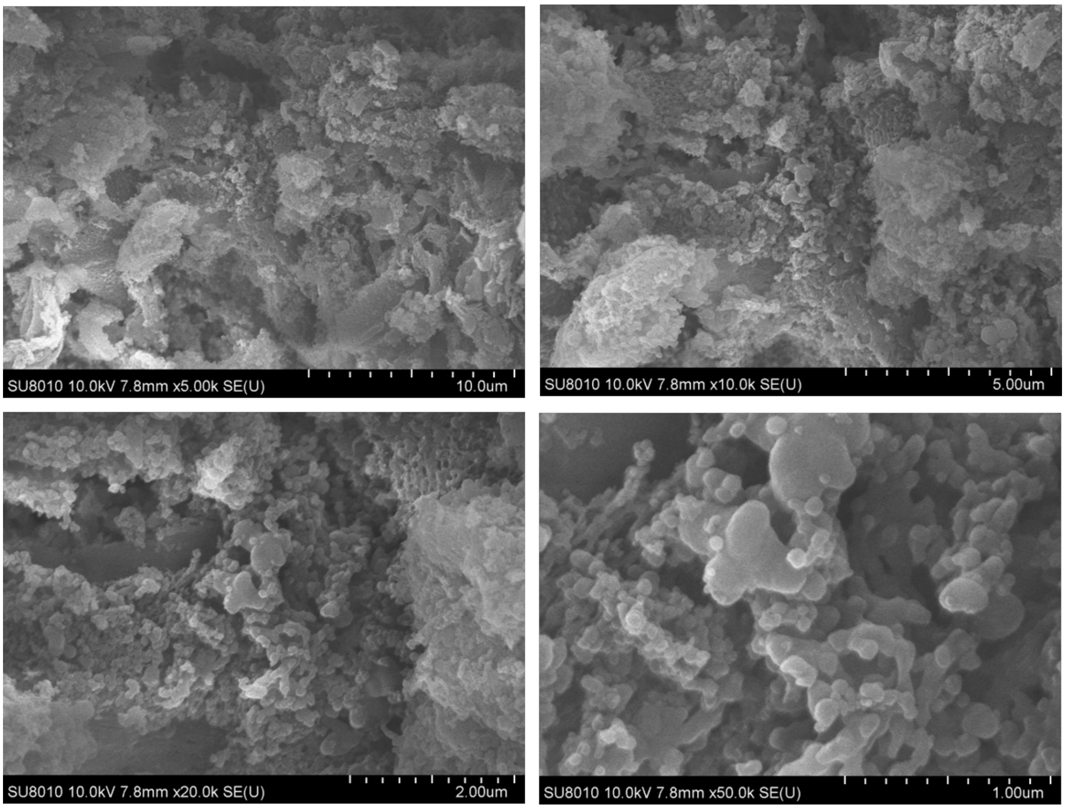

Supplementary Fig. 17 Morphologies of polymers. SEM images of P-HEG-5.

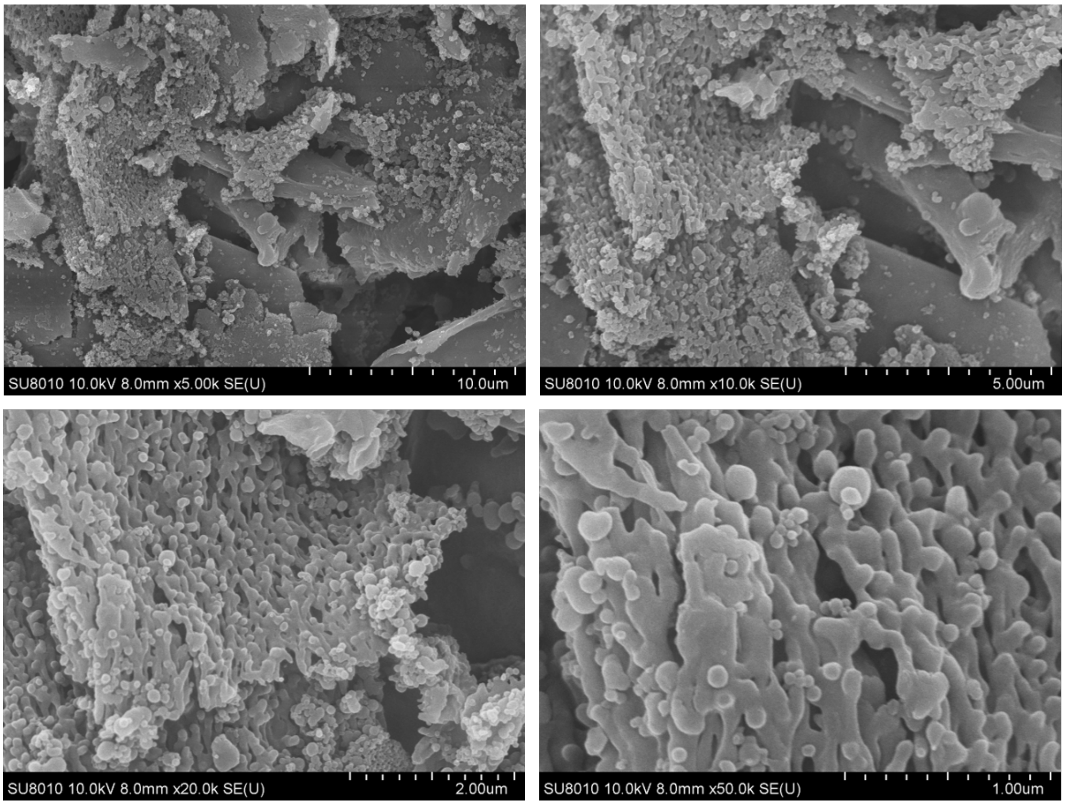

Supplementary Fig. 18 Morphologies of polymers. SEM images of P-HEG-10.

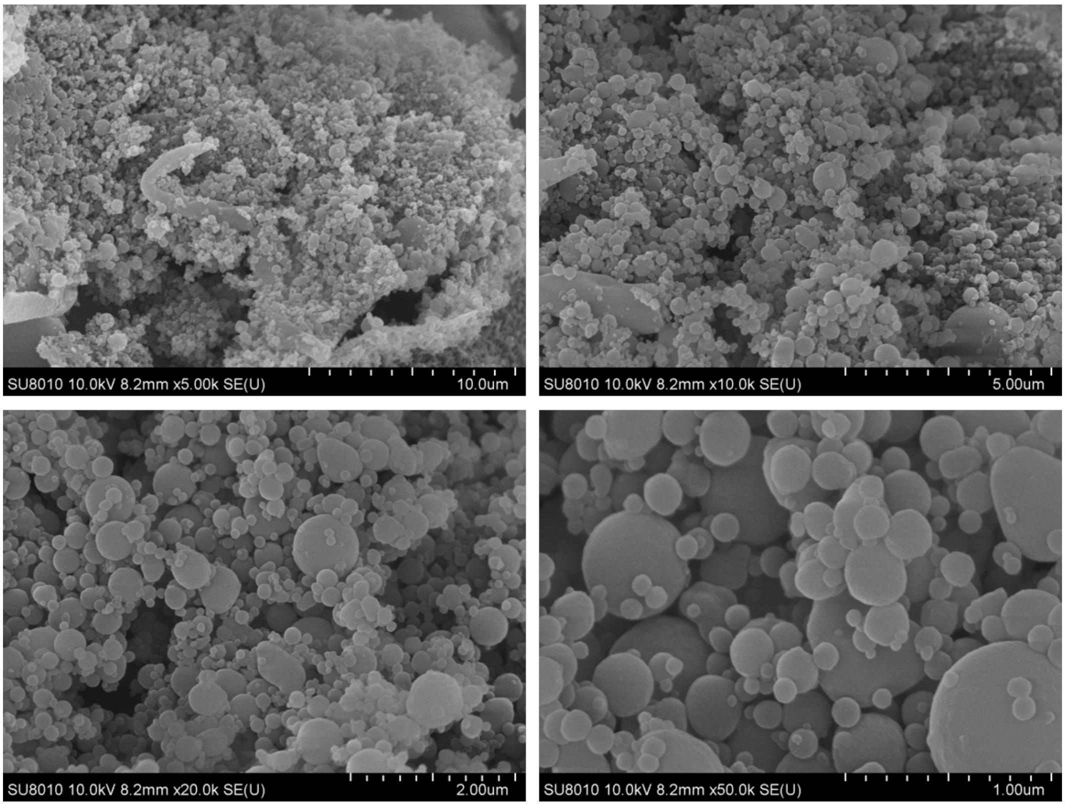

Supplementary Fig. 19 Morphologies of polymers. SEM images of P-HEG-20.

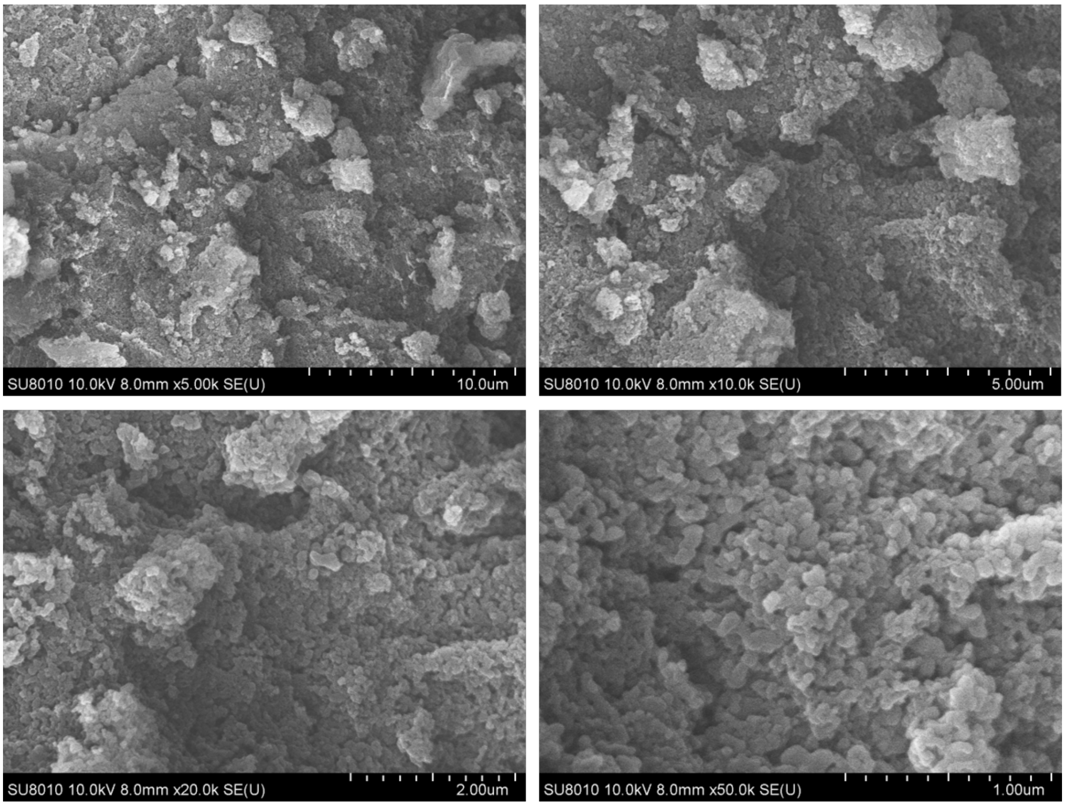

Supplementary Fig. 20 Morphologies of polymers. SEM images of P-EA-5.

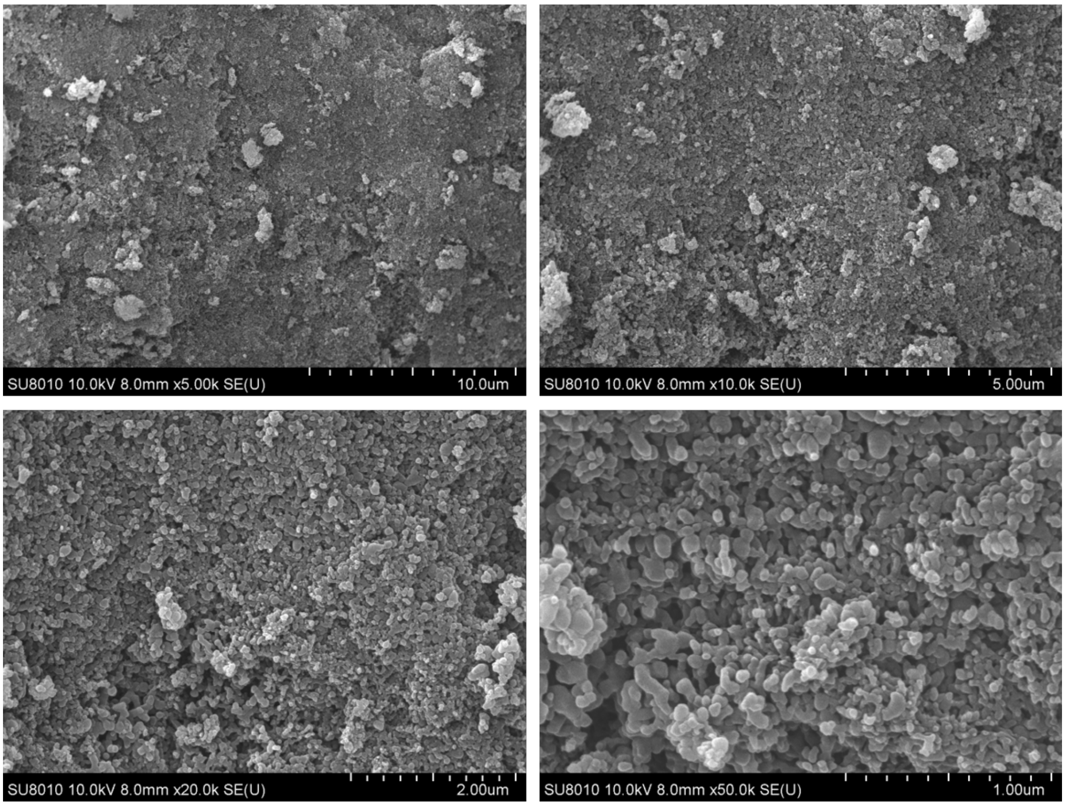

Supplementary Fig. 21 Morphologies of polymers. SEM images of P-EA-10.

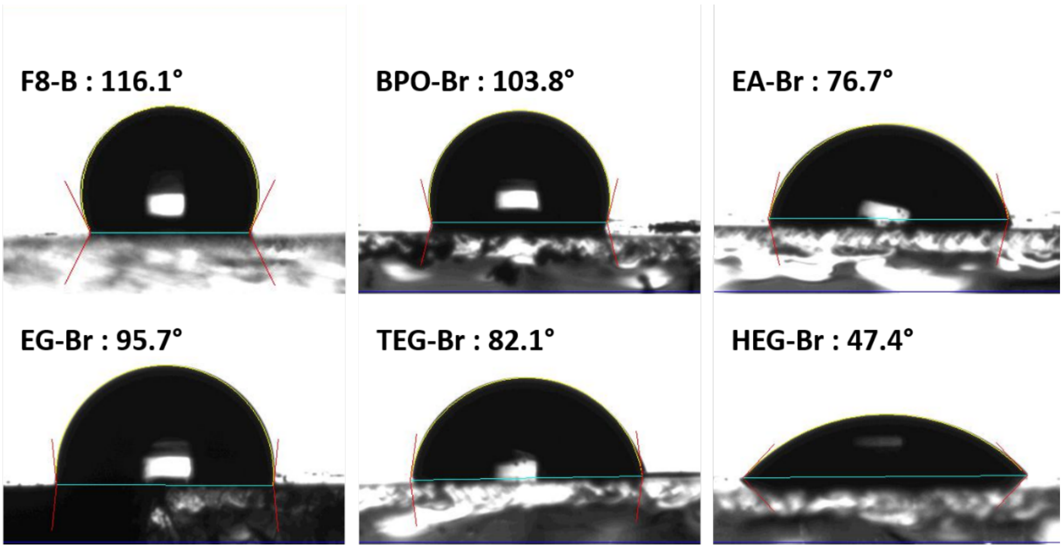

Supplementary Fig. 22 Hydrophilicity of co-monomers. Water contact angle images of the monomers.

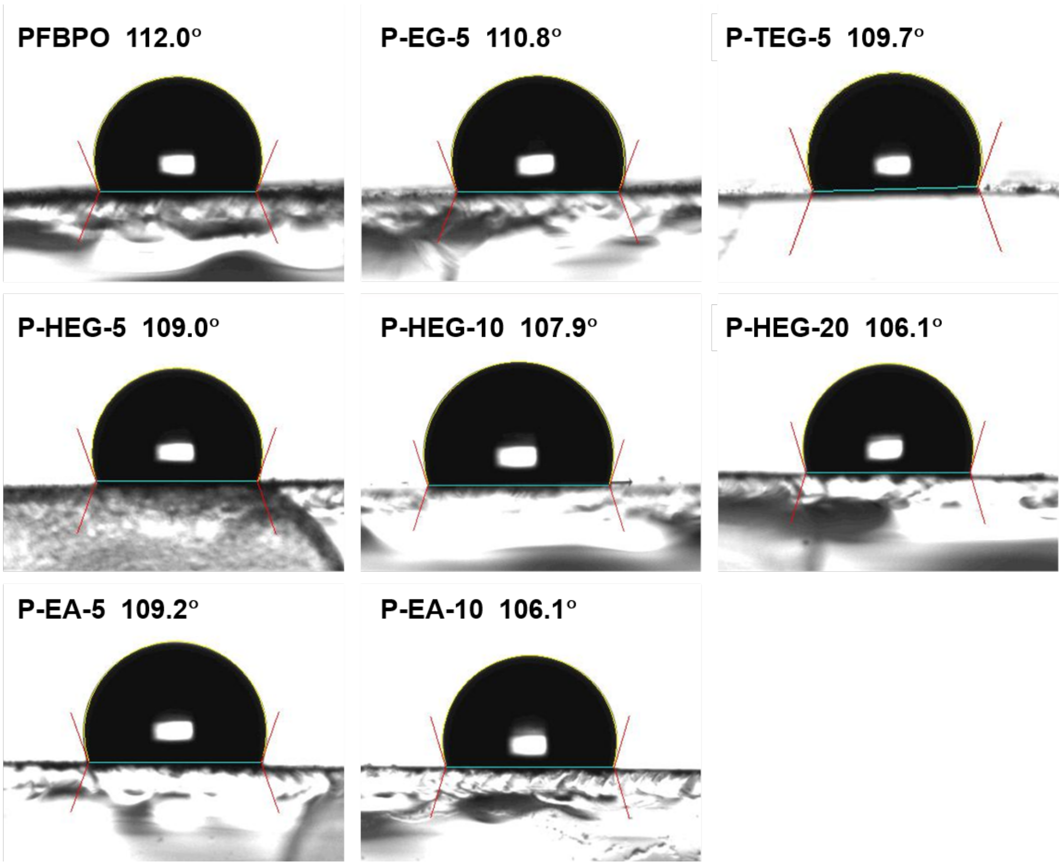

Supplementary Fig. 23 Hydrophilicity of polymers. Water contact angle images of the polymers.

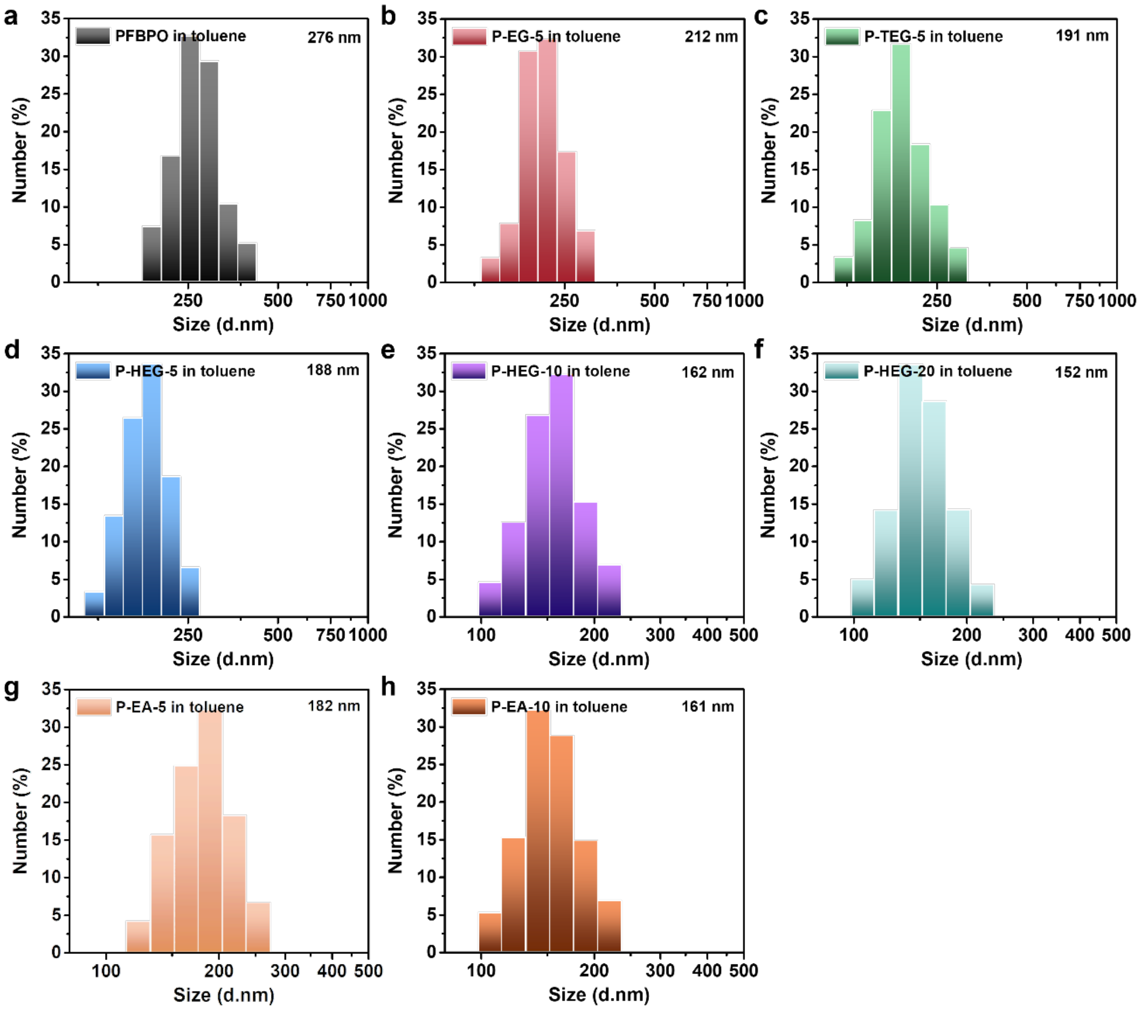

Supplementary Fig. 24 DLS analysis. DLS hydrodynamic diameters of **a** PFBPO, **b** P-EG-5, **c** P-TEG-5, **d** P-HEG-5, **e** P-HEG-10, **f** P-HEG-20, **g** P-EA-5, and **h** P-EA-10 were determined in toluene.

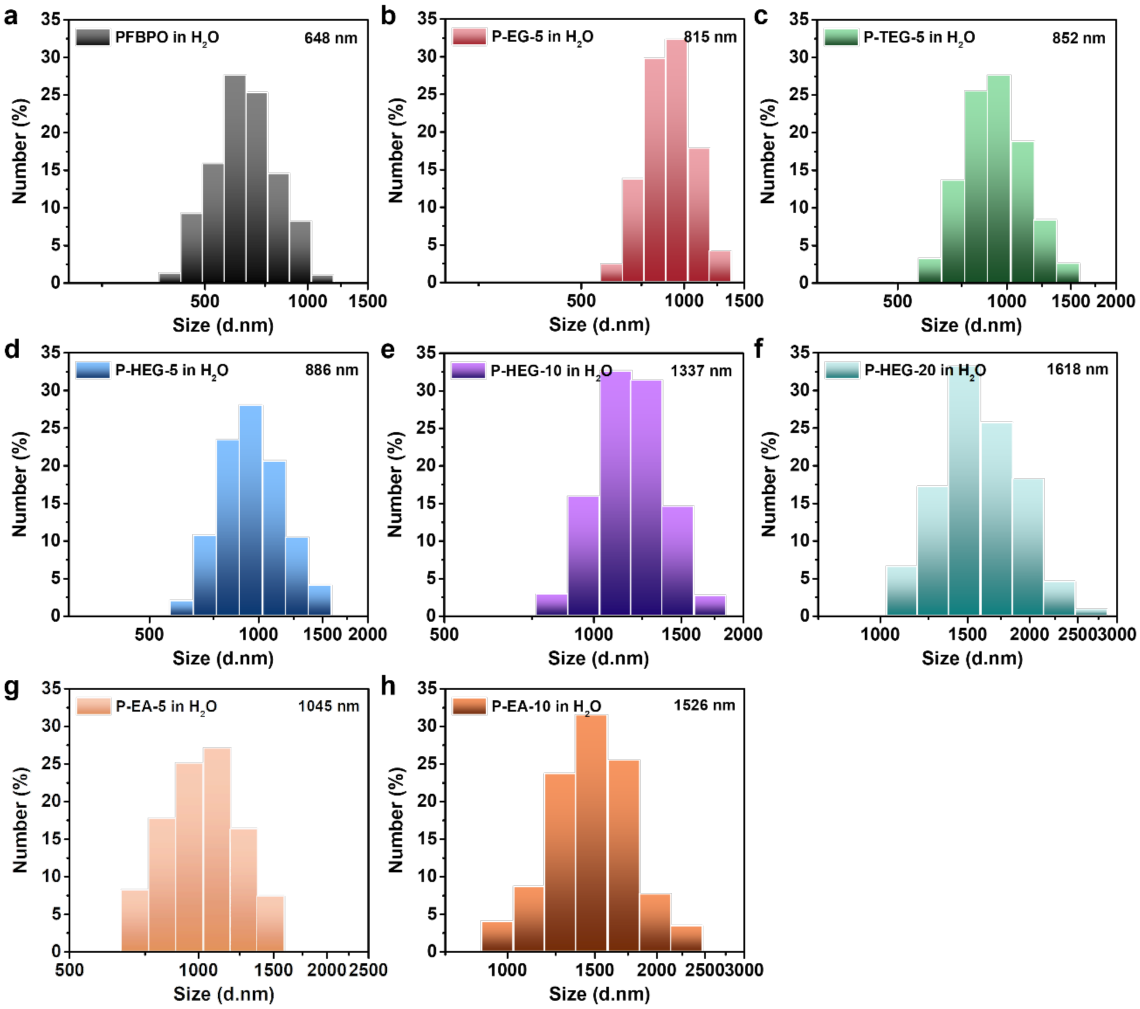

Supplementary Fig. 25 DLS analysis. DLS hydrodynamic diameters of **a** PFBPO, **b** P-EG-5, **c** P-TEG-5, **d** P-HEG-5, **e** P-HEG-10, **f** P-HEG-20, **g** P-EA-5, and **h** P-EA-10 were determined in pure water.

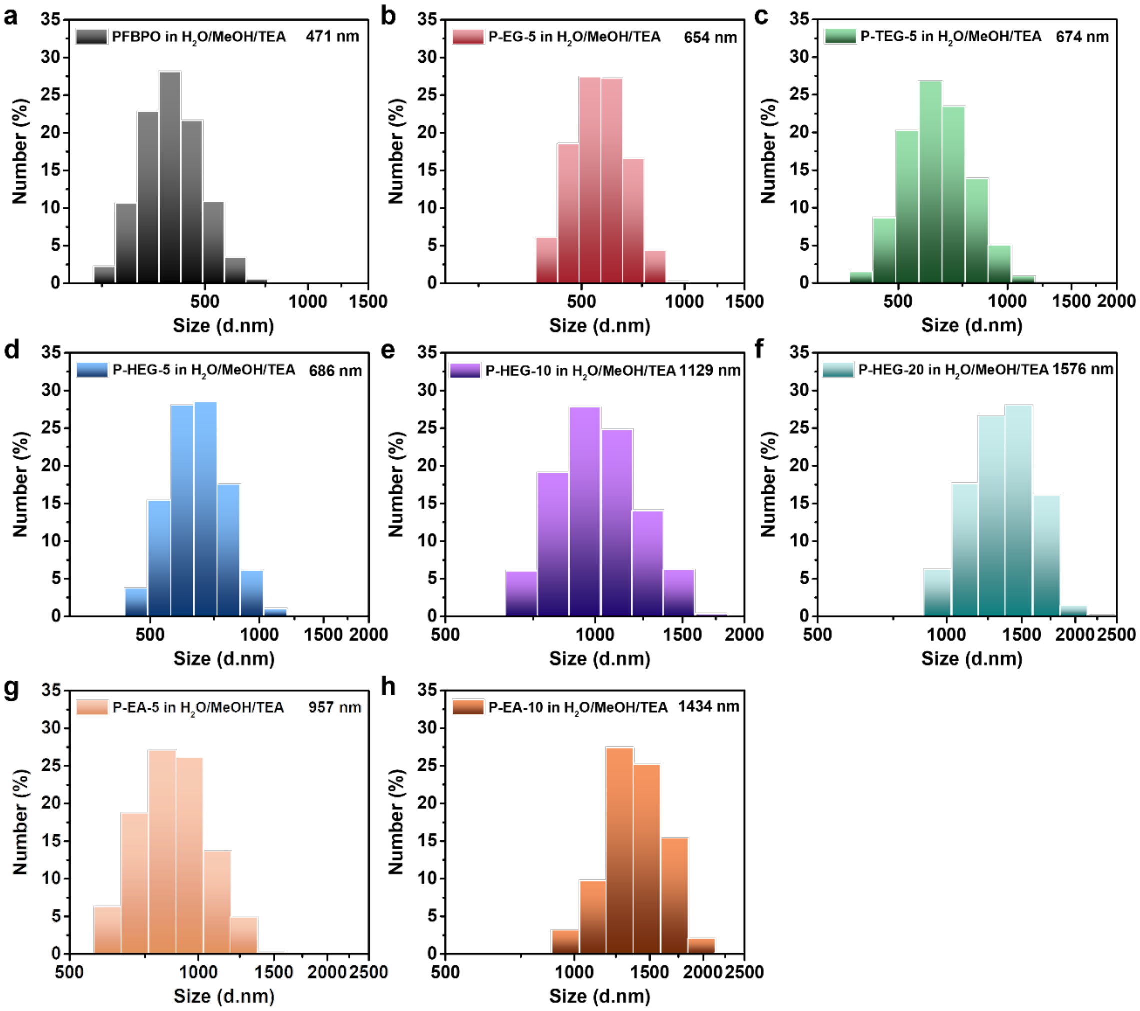

Polymer	Size in pure toluene (nm)	Size in H_2O/MeOH/TEA (nm)	Size in pure water (nm)
**PFBPO**	276	471	648
**P-EG-5**	212	654	815
**P-TEG-5**	191	674	852
**P-HEG-5**	188	686	886
**P-HEG-10**	162	1129	1337
**P-HEG-20**	152	1576	1618
**P-EA-5**	182	957	1045
**P-EA-10**	161	1434	1526

Supplementary Fig. 26 DLS analysis. DLS hydrodynamic diameters of **a** PFBPO, **b** P-EG-5, **c** P-TEG-5, **d** P-HEG-5, **e** P-HEG-10, **f** P-HEG-20, **g** P-EA-5, and **h** P-EA-10 were determined in a mixture solution consisting of equal volumes of H_2O, MeOH, and TEA.

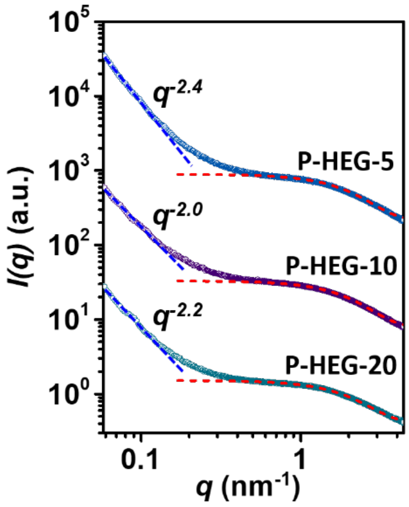

Supplementary Fig. 27 SAXS analysis. SAXS patterns of P-5HEG, P-10HEG, and P-20HEG polymers in solution mixture consisting of equal volumes of H_2O, MeOH, and TEA.

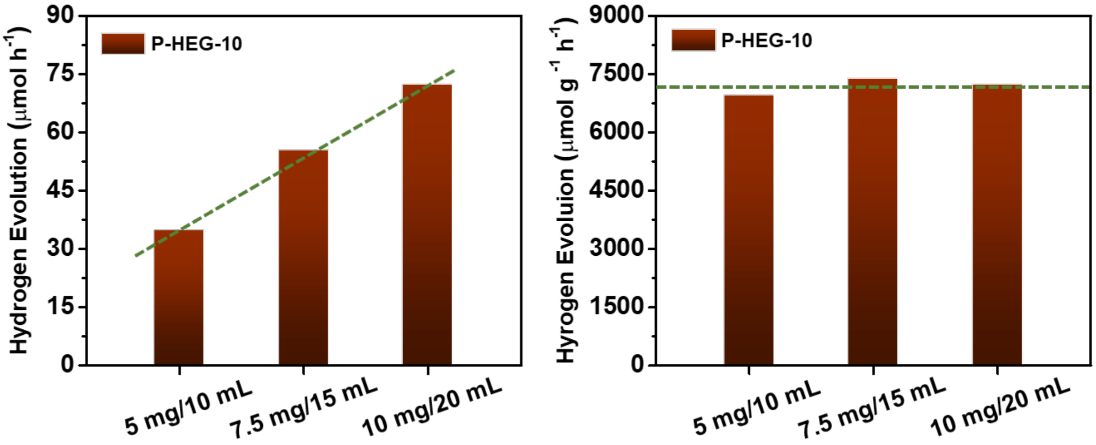

Supplementary Fig. 28 Photocatalytic hydrogen evolution. Effect of the quantity of photocatalytic solution on the HER of P-10HEG.

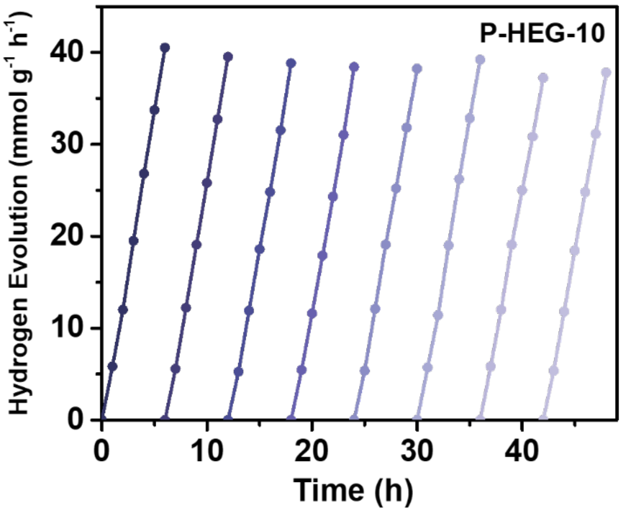

Supplementary Fig. 29 Photocatalytic hydrogen evolution. Long-term photocatalytic cycling up to 48 h was measured to test the durability of P-HEG-10.

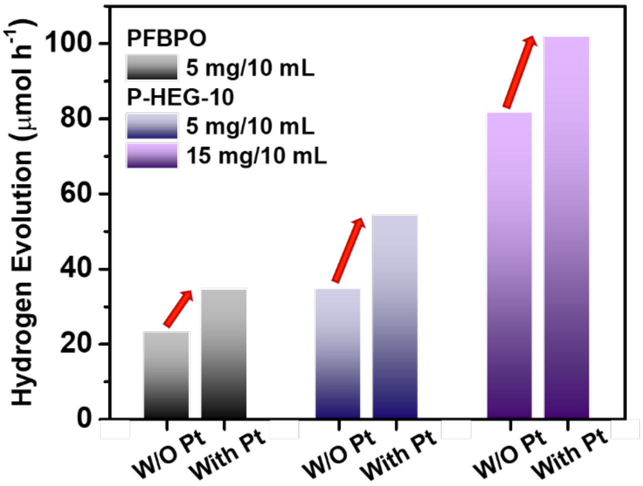

Supplementary Fig. 30 Photocatalytic hydrogen evolution. Influence of the Pt co-catalyst on the HER of P-HEG-10 and PFBPO.

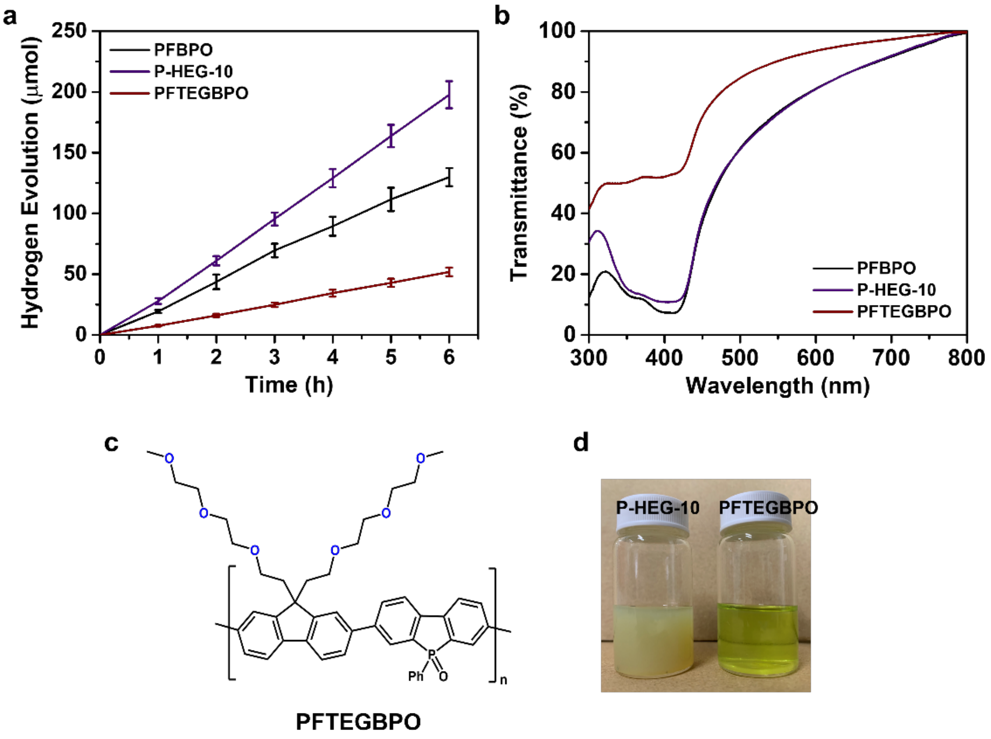

Supplementary Fig. 31 Applicability analysis. Comparison of applicability of main-chain-engineered DCP and side-chain-engineered CP. **a** Time-dependent HER under standard photocatalytic condition (5 mg photocatalyst in 10 mL solution mixture consisting of equal volumes of H_2O, MeOH, and TEA), **b** Transmission spectra under otherwise identical conditions with hydrogen evolution experiments. **c** Molecular structure of side-chain-engineered PFTEGBPO. **d** Images of P-HEG-10 and PFTEGBPO photocatalytic solutions.

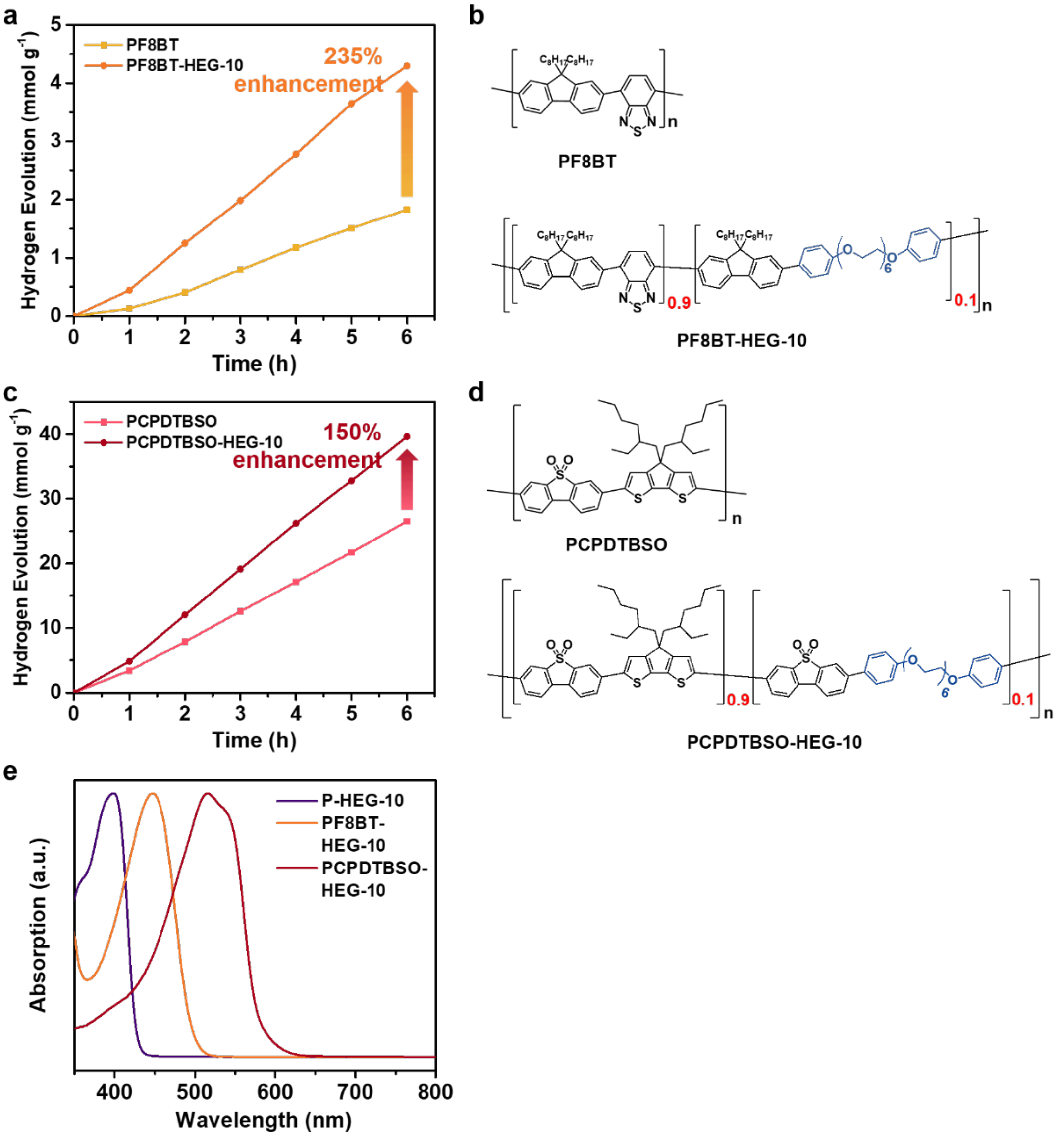

Supplementary Fig. 32 Universality analysis. The main-chain engineering approach was further applied on polymers with different absorption maxima, PF8BT ($\lambda_{max.}$ = 450 nm) and PCPDTBSO ($\lambda_{max.}$ = 520 nm), to demonstrate that our approach is novel and universal. **a** Time-dependent HER of PF8BT and PF8BT-HEG-10 under standard photocatalytic condition (5 mg photocatalyst in 10 mL solution mixture consisting of equal volumes of H_2O, MeOH, and TEA). **b** Molecular structure of PF8BT and PF8BT-HEG-10. **c** Time-dependent HER of PCPDTBSO, and PCPDTBSO-HEG-10 under standard photocatalytic condition. **d** Molecular structure of PCPDTBSO and PCPDTBSO-HEG-10. **e** Absorption spectra of polymers with different absorption maxima, PFBPO, PF8BT and PCPDTBSO.

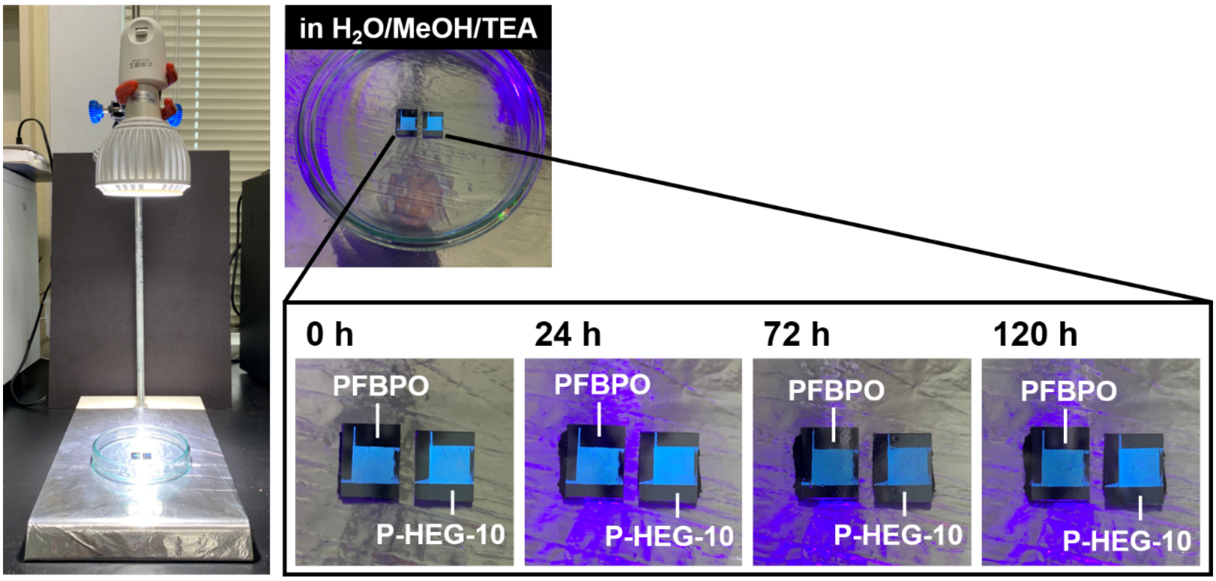

Supplementary Fig. 33 Stability analysis of polymer films. The PFBPO and P-HEG-10 thin-films were directly soaked in photocatalytic solution (the volume ratio of water, TEA, and methanol is 1:1:1) under light illustration for over 120 hours to examine their photocatalytic stability.

Supplementary Table 1 Physical properties of various polymers.

Polymer	Emission (nm)	M_n[a]	M_w[a]	PDI (M_w/M_n)[a]	T_d (°C)[b]	Pd (wt %)[c]	HER (μmol h^{-1}) (5 mg/10 mL)/(10 mg/10 mL)/(15 mg/10 mL)
PFBPO	456	10,435	16,070	1.54	419	0.037	23.0 (36.5)[d]/31.0/37.3
P-EG-5	454	12,975	18,165	1.40	420	0.139	-
P-TEG-5	455	12,839	18,616	1.45	422	0.209	-
P-HEG-5	456	14,089	20,288	1.44	420	0.069	-
P-HEG-10	458	13,059	20,502	1.57	423	0.024	34.7 (54.1)[d]/66.1/ 81.6 (101.9)[d]
P-HEG-20	456	13,415	20,258	1.51	406	0.017	20.2/39.5/60.8
P-EA-5	454	15,155	21,975	1.45	421	0.135	-
P-EA-10	457	13,851	21,192	1.53	414	0.093	-

[a]M_w, M_n, polydispersity index (PDI) were determined by gel permeation chromatography in THF at 40 °C. [b]The decomposition temperature was determined by thermogravimetric analysis. [c]The residual Pd contents were determined by ICP-MS. [d]HER measurements were performed in with 5 wt.% Pt co-catalyst.

Supplementary Table 2 Comparison of the HERs and AQYs of various photocatalysts under solution state photocatalytic systems in the literature.

Polymer Catalysts	Metal Co-Catalysts	HER (mmol $h^{-1}g^{-1}$)	AQY (%)	Conditions	References
**Solution systems**					
P10	Pd-residue	3.26 (> 420 nm)	11.6 @ 420 nm	H_2O/MeOH/TEA	*Nat. Commun.* **9**, 4968 (2018)
CTF-1-100W	3 wt.% Pt	5.50 (AM 1.5)	6.0 @ 420 nm<br>3.6 @ 460 nm	H_2O/TEOA/MeOH	*Energy Environ. Sci.* **11**, 1617 (2018)
FS-COF+WS5F	8 wt.% Pt	16.3(> 420 nm)	7.3 @ 420 nm	H_2O/Ascorbic Acid (0.1 M)	*Nat. Chem.* **10**, 1180 (2018)
PBDTBT-7EO	3 wt.% Pt	12.8 (> 420 nm)	0.13 @ 420 nm<br>0.14 @ 450 nm	H_2O/Ascorbic Acid (0.2 M)	*iScience* **13**, 33 (2019)
PDPP3B-O4	1 wt.% Pt	5.53 (> 400 nm)	0.84 @ 420 nm<br>5.76 @ 450 nm	H_2O/TEOA	*Polym. Chem.* **10**, 6473 (2019)
P7O-4N	3 wt.% Pt	1.56	-	H_2O/Ascorbic Acid (0.2 M)	*Chem. J. Chinese U.* (2019), DOI: 10.7503/cjcu20190647
BBT-SC2NH2	Pd-residue	9.06 (> 420 nm)	3.3 @ 420 nm	H_2O/TEOA	*J. Mater. Chem. A* **7**, 16277 (2019)
Tr-F3N	Pd-residue	0.538 (> 300 nm)	-	H_2O/TEOA/ ethylene glycol	*J. Mater. Chem. A* **7**, 19087 (2019)
P64	Pd-residue	3.53 (AM 1.5)	20.7 @ 420 nm	H_2O/MeOH/TEA	*J. Am. Chem. Soc.* **141**, 9063 (2019)
P62	Pd-residue	3.50 (AM 1.5)	15.1 @ 420 nm	H_2O/MeOH/TEA	
BBT-FC8O5	Pd-residue	10.4 (> 420 nm)	2.4 @ 420 nm<br>0.8 @ 450 nm	H_2O/TEOA	*Appl. Surf. Energy* **499**, 143865 (2020)
PS-TEG	Pd-residue	2.9 (> 420 nm)	-	H_2O/MeOH/TEA	*Energy Environ. Sci.* **13**, 1843 (2020)
PTB7-Th/EH-IDTBR	10 wt.% Pt	64.43 (350-800 nm)	6.2 @ 700 nm	H_2O/Ascorbic Acid (0.2 M)	*Nat. Mater.* **19**, 559 (2020)
1% wNCQDs/ PFTBTA Pdots	Pd-residue	4.49 (> 420 nm)	0.56 @ 420 nm	H_2O/AA	*Appl. Catal. B* **283**, 119659 (2021)
DPBT-TP	7.5 wt.% Pt	17.8 (380-780 nm)	3.28 @ 420 nm<br>0.60 @ 500 nm	H_2O/MeOH/TEA	*Appl. Catal. B* **285**, 119802 (2021)
**P-HEG-10**	5 wt.% Pt	**10.8 (380-780 nm)**	**18.19 @ 420 nm**<br>**17.82 @ 460 nm**	**H_2O/MeOH/TEA**	**This work**

Supplementary Table 3 Comparison of the HERs of various photocatalysts under thin-film state photocatalytic systems in the literature.

Polymer Catalysts	Metal Co-Catalysts	HER (mmol $h^{-1}g^{-1}$)	Conditions	References
**Thin film systems**				
FS-COF+WS5F	8 wt.% Pt	Single dropcast cycle: 15.8 mmol m^{-2} h^{-1} (> 420 nm)	H_2O/Ascorbic Acid (0.1 M)	*Nat. Chem.* **10**, 1180 (2018)
PS-TEG	Pd-residue	Three slides stacking in series: 6.4 mmol m^{-2} h^{-1} (> 420 nm)	H_2O/MeOH/TEA	*Energy Environ. Sci.* **13**, 1843 (2020)
**P-HEG-10**	**Pd-residue**	**Single dropcast cycle: 16.6 mmol m^{-2} h^{-1} (380-780 nm)**	**H_2O/MeOH/TEA**	**This work**
